# Can detailed instructions and comprehension checks increase the validity of crosswise model estimates?

**DOI:** 10.1371/journal.pone.0235403

**Published:** 2020-06-30

**Authors:** Julia Meisters, Adrian Hoffmann, Jochen Musch

**Affiliations:** Department of Experimental Psychology, University of Duesseldorf, Duesseldorf, Germany; University of Copenhagen, DENMARK

## Abstract

The crosswise model is an indirect questioning technique designed to control for socially desirable responding. Although the technique has delivered promising results in terms of improved validity in survey studies of sensitive issues, recent studies have indicated that the crosswise model may sometimes produce false positives. Hence, we investigated whether an insufficient understanding of the crosswise model instructions might be responsible for these false positives and whether ensuring a deeper understanding of the model and surveying more highly educated respondents reduces the problem of false positives. To this end, we experimentally manipulated the amount of information respondents received in the crosswise model instructions. We compared a crosswise model condition with only brief instructions and a crosswise model condition with detailed instructions and additional comprehension checks. Additionally, we compared the validity of crosswise model estimates between a higher- and a lower-educated subgroup of respondents. Our results indicate that false positives among highly educated respondents can be reduced when detailed instructions and comprehension checks are employed. Since false positives can also occur in direct questioning, they do not appear to be a specific flaw of the crosswise model, but rather a more general problem of self-reports on sensitive topics. False negatives were found to occur for all questioning techniques, but were less prevalent in the crosswise model than in the direct questioning condition. We highlight the importance of comprehension checks when applying indirect questioning and emphasize the necessity of developing instructions suitable for lower-educated respondents.

## Introduction

Direct self-reports on sensitive personal attributes are susceptible to socially desirable responding. Specifically, some respondents may respond in line with social norms, rather than truthfully, leading to an overestimation of the prevalence of socially desirable and an underestimation of the prevalence of socially undesirable attributes. This threatens the validity of direct self-reports [1–3].

Indirect questioning techniques such as the randomized response technique (RRT [4]) have been proposed to control for social desirability bias. In the original RRT, respondents are presented with two statements: a sensitive statement A (e.g. *I have used cocaine*) and its opposite B (*I have never used cocaine*). Respondents are instructed to employ a randomization procedure, e.g. throwing a die, whose outcome is only known to the respondent, but concealed from the interviewer. Depending on the outcome of this randomization procedure, respondents are asked to respond to either statement A or statement B by indicating whether the respective statement is “true” or “false”. Since the interviewer does not know which statement an answer refers to, respondents’ privacy is protected. However, the distribution of randomization outcomes is known; therefore, the proportion of respondents carrying the sensitive attribute can be deduced on the sample level.

So-called “weak” validation studies compare prevalence estimates obtained via RRTs with prevalence estimates obtained via a direct question. A meta-analysis of 32 “weak” validation studies [5] found that RRTs generally lead to higher and thus presumably more valid prevalence estimates than direct questioning (DQ). However, the “more-is-better” criterion employed in weak validation studies does not allow definite conclusions to be drawn regarding the validity of RRTs. Rather, definite conclusions result from “strong” validation studies, in which prevalence estimates obtained via RRTs are compared with the ground truth, that is, the known prevalence of a sensitive attribute in a given sample [6]. A meta-analysis of 6 strong validation studies [5] found that RRTs are more valid than DQ, especially when the topic under investigation is highly sensitive; however, RRTs still notably underestimated known prevalences. Moreover, because they add random noise to the estimator, RRTs are generally less efficient than DQ [7]. Therefore, the application of RRTs is only justified when the topic under investigation is sensitive in nature and an RRT can help to avoid response distortions due to socially desirable responding [5].

### The crosswise model: A promising alternative to conventional RRT

**Fig 1:**
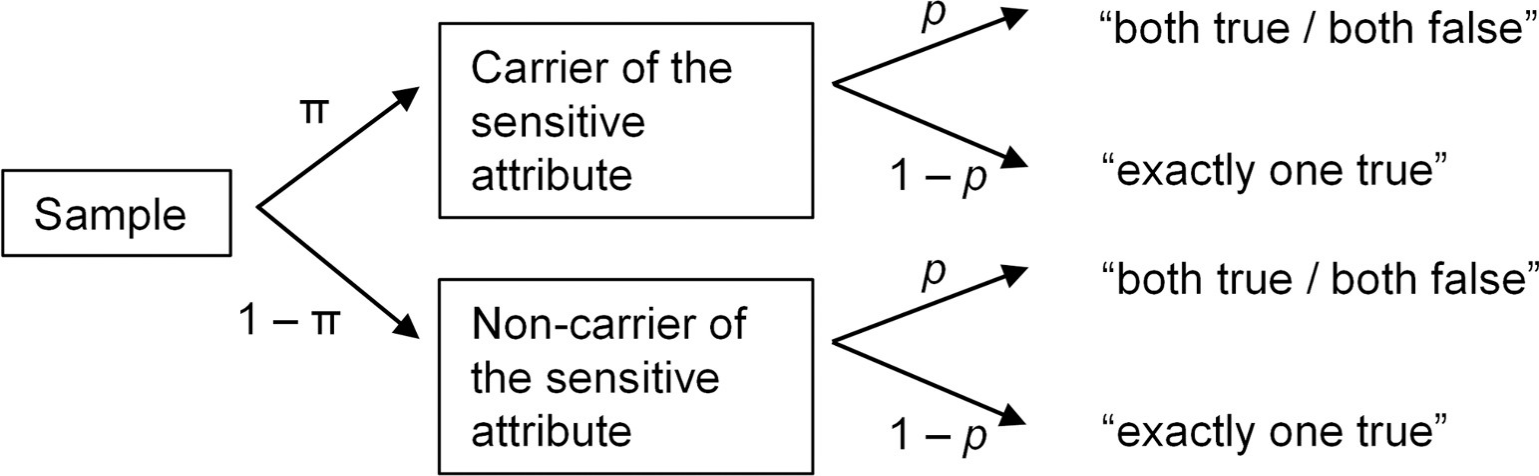
Tree diagram of the crosswise model. The parameter π represents the unknown prevalence of the sensitive attribute, and the parameter *p* represents the known randomization probability.

Nonrandomized response techniques [8, 9], such as the crosswise model (CWM), represent recent advancements of the RRT. Questions in nonrandomized response format do not require an external randomization device and employ simpler instructions, supposedly making them easier to administer for the experimenter and easier to understand for the respondents. In the CWM, respondents are presented with two statements–a sensitive statement A (e.g. *I have used cocaine*) with unknown prevalence and a non-sensitive statement B (e.g. *I was born in November or December*) serving as a randomization device. Respondents are instructed to give a joint answer to these questions indicating whether “both statements are true or both statements are false” or whether “exactly one of the statements (irrespective of which one) is true”. Fig 1 shows the CWM as a tree diagram. Significantly higher and thus presumably more valid prevalence estimates have been obtained via the CWM as compared to DQ for sensitive attributes such as xenophobia [10, 11], plagiarism [12], tax evasion [13, 14], distrust in the Trust Game [14], crossing the street on a red light in plain view of children [15], the use of anabolic steroids among bodybuilders [16], intention to vote for the far-right German party Alternative for Germany [17], and prejudice against female leaders [18]. Moreover, in one strong validation study, the CWM accurately estimated the prevalence of experimentally induced cheating behavior, while DQ led to a severe underestimation [19]. Furthermore, the CWM is easier to understand than other RRT models and is perceived as significantly more confidential than DQ [20].

### Cautionary evidence of false positives in the CWM

However, the results of two recent studies by Höglinger and Diekmann [21] and Höglinger and Jann [22] indicate that the CWM may sometimes produce false positives, that is, some non-carriers of the sensitive attribute are falsely classified as carriers. Höglinger and Diekmann [21] asked respondents whether they had ever received a donated organ and whether they had ever suffered from Chagas disease, both of which are attributes with a prevalence close to zero. As expected, DQ provided estimates that did not significantly differ from zero. In the CWM condition, however, the prevalence estimates for the two zero-prevalence items–and thus false positive rates–were 8% and 5%, respectively. In an additional individual-level validation, the authors asked about a somewhat sensitive control attribute (i.e. whether respondents had completed the German general university entrance qualification). Again, DQ provided a prevalence estimate close to zero; for the CWM, a false positive rate of 7% was observed. Remarkably, the CWM also produced a substantial number of false negatives, that is, some carriers of the sensitive attribute were falsely classified as non-carriers. As the false positives and false negatives cancelled each other out on the aggregate level, the overall prevalence estimates accurately reflected the known prevalence. However, the interpretability of this individual-level validation is limited because the relevant question was presented as a practice question in the CWM but not in the DQ condition, and because the prevalence estimates were compared with an external criterion that had been collected up to five years earlier and in a different response format. Finally, the authors found that the rate of false positives was moderated by the choice of the unrelated questions used for randomization. This finding implies that researchers using indirect questioning techniques must make a well-informed decision about which unrelated question to use.

In the second study, Höglinger and Jann [22] conducted individual-level validations via an online experiment in which participants had to play one of two dice games: In the *prediction game*, they had to predict the outcome of a die roll in private and were then asked to roll the die. Afterwards, to determine whether they qualified for a payout, respondents were asked to indicate whether they had rolled the predicted outcome. Since the predictions were made in private, cheating was observable only on the group level; an individual-level validation could only be computed by making two strong assumptions. First, it had to be assumed that all respondents whose predictions were correct actually claimed the payout; second, the false positive rate among respondents whose predictions were correct and who claimed the payout had to be assumed to be equal to the false positive rate among respondents whose predictions were incorrect and who did not claim the payout. In the *roll-a-six game*, participants had to roll a die and were then asked to indicate whether they had rolled a six, in which case they would receive a financial reward. In this second game, the outcomes were tracked, making cheating directly observable on the individual level. After each of the two dice games, participants had to answer a sensitive question about whether they had cheated in the respective game. On the aggregate level, the CWM estimates of cheating were significantly higher than the DQ estimates for both games, thus satisfying the “more-is-better” criterion. However, in both individual-level validations, the CWM produced more than 10% false positives, whereas the false positive rate in the DQ condition did not significantly differ from zero.

At this point, it is not yet understood whether false positives only occur under certain circumstances, or whether they pose a general threat to the validity of the CWM and of indirect questioning techniques as a whole. Höglinger and Diekmann [21] exploratively examined potential causes and correlates of false positives, but did not find a consistent pattern. Respondents who sped through the CWM instructions and may therefore not have understood them properly produced descriptively, but not significantly, more false positives. However, the reverse pattern emerged when only the sensitive questions were examined: here, speeders tended to produce fewer false positives. The authors hypothesized that the problem of false positives might be less severe in “better designed C[W]M implementations” (p. 5). Consequently, identifying conditions under which respondents show high levels of understanding and trust in the method could help to improve CWM implementation. Trust and understanding are necessary prerequisites for RRTs to yield valid results [23], but are often not achieved [20, 23–28]. Although the comprehensibility of the CWM, operationalized in terms of correct responses to scenario-based questions testing understanding of the model, was shown to be comparatively higher than the comprehensibility of other indirect questioning techniques, more than 16% incorrect responses were still observed [20]. Accordingly, Hoffmann et al. [20] suggested employing detailed instructions and comprehension checks to ensure respondents’ understanding of and trust in indirect questioning techniques. Building upon these recommendations, the present study sought to investigate whether the validity of results obtained via the CWM can be improved by providing respondents with more detailed instructions.

### The present study

We sought to obtain a deeper understanding of the conditions under which false positives and false negatives occur in CWM surveys, and how they affect measurement validity. To this end, we conducted a strong validation based on a known external criterion by employing the anagram paradigm introduced by Hoffmann et al. [19]. This paradigm induces cheating to generate a sensitive attribute with known prevalence in the sample. It allowed us to compare all prevalence estimates with a known true value, and to conduct separate analyses of false negatives among carriers and false positives among non-carriers of the sensitive attribute. Based on the results of Höglinger and Diekmann [21] and Höglinger and Jann [22], we hypothesized that prevalence estimates based on self-reports would suffer from both false positives and false negatives. Moreover, we expected false positives to occur more frequently in the CWM condition compared to the DQ condition [cf. 21, 22]. In contrast, we expected false negatives to occur more frequently in the DQ condition compared to the CWM condition due to the influence of socially desirable responding [cf. 10, 12, 13].

Most importantly, the current study sought to identify potential means of reducing false positives and false negatives in order to maximize the validity of prevalence estimates obtained via indirect questioning techniques such as the CWM. We therefore tested the assumption that an insufficient understanding of and trust in the method are major causes of false positives in the CWM. To this end, we experimentally manipulated the amount of information respondents received in the CWM instructions. Specifically, we compared two groups, one of which received detailed instructions combined with several questions assessing comprehension (CWM detailed), and the other of which received only brief instructions and no comprehension questions (CWM brief). We expected that false positives were less likely when respondents had a better understanding of the CWM (CWM detailed) than when they had only a superficial understanding of the method (CWM brief). Regarding the rate of false negatives, we did not have a directed hypothesis. On the one hand, it might seem reasonable to expect that a better understanding of the method reduces false negatives resulting from misunderstandings; on the other hand, a better understanding may also help respondents to present themselves as non-carriers, which in turn could increase false negatives.

Since comprehension of CWM instructions has been shown to be positively associated with education [20], and lower-educated respondents have been found to disobey RRT instructions more often [29], we additionally compared the false positive and false negative rates between a higher-educated (at least 12 years of education, the German *Abitur*) and a lower-educated subgroup (at most 10 years of education, the German *Realschule*). We expected a higher false positive rate among lower educated than among highly educated respondents.

## Methods

### Participants

Respondents were recruited by a commercial German online panel provider. To avoid a lack of understanding of the instructions due to language difficulties, a necessary prerequisite for participation was that respondents were German native speakers. Moreover, to avoid confounding education with age, we restricted the age range of respondents to 30 to 40 years. This homogeneity with respect to age helped maximize the statistical power for testing our main hypotheses because it reduced the variance in education that would have been present in a more age-diverse sample due to a general trend towards higher educational attainment among younger cohorts in Germany [30].

The survey was carried out in accordance with the revised Declaration of Helsinki [31] and the ethical guidelines of the German Society for Psychology [32]. In Germany, there is no binding obligation that research projects can only be carried out after approval by an ethics committee. Participation in the present study could not have any negative consequences for the respondents, and anonymity was ensured at all times. The respondents participated voluntarily and after informed consent was obtained. There was no risk that participation could cause any physical or mental damage or discomfort to participants beyond their normal everyday experiences. Therefore, ethics committee approval was not required according to the “Ethical Research Principles and Test Methods in the Social and Economic Sciences” formulated by the Ethics Research Working Group of the German Data Forum [33] and the “Ethical Recommendations of the German Psychological Society” [34].

Sample size was determined on the basis of a priori power considerations indicating that to ensure sufficient statistical power (1-ß ≥ .80), a sample of more than 1500 participants was required. We decided to allocate twice as many respondents to the CWM conditions than to the DQ condition to compensate for the lower efficiency of the CWM that is a consequence of the randomization procedure [7, 35].

The initial sample consisted of 3060 respondents, with an equal distribution regarding education (higher-educated: at least 12 years of education, the German *Abitur*; lower-educated: at most 10 years of education, the German *Realschule*) and gender (male vs female). Due to incomplete data, 347 respondents had to be excluded from the analysis (11.34% of the initial sample). This dropout was nonselective in terms of cheating on the anagram task, χ²(1, *N* = 2934) = 2.75, *p* = .098, *Cramer’s V* = .03. Dropout rates were slightly lower among higher-educated respondents (8.25%) compared with lower-educated respondents (13.13%). χ²(1, *N* = 3040) = 18.87, *p* < .001, *Cramer’s V* = .08. However, this effect was small and thus considered negligible. Respondents in the CWM detailed condition were more likely to drop out (19.20%) than respondents in the other conditions (CWM brief: 3.00%; DQ: 3.67%), χ²(2, *N* = 3002) = 211.75, *p* < .001, *Cramer’s V* = .27.

The final sample consisted of 2713 respondents (50.31% female) with a mean age of *M* = 34.73 years (*SD* = 3.15). Half of the respondents (49.98%) were lower-educated, while the other half were higher-educated (50.02%). Overall, 972 respondents (35.83%) were assigned to the CWM detailed condition, 1164 (42.90%) to the CWM brief condition, and 577 (21.27%) to the DQ condition. Respondents in the three conditions did not differ with regard to education, χ²(2) = 0.92, *p* = .632, *Cramer’s V* = .02.

### Measures

#### Anagram cheating task

To enable a strong validation, we experimentally induced a sensitive attribute with known prevalence in the sample using the anagram paradigm established by Hoffmann et al. [19]. This paradigm consists of two parts: the anagram task itself and a subsequent opportunity for respondents to overreport their performance–that is, to cheat on the task. In the first part of the anagram task, respondents are presented with three scrambled words (“anagrams”). Instead of directly reporting the solutions to these anagrams, respondents are instructed to solve the anagrams in their head. The anagrams are presented for a maximum of 20 seconds each; respondents can continue to the next anagram anytime by pressing a button. Unknown to the respondents, the first two anagrams are very easy to solve (solved by > 99% of the respondents in a pilot study [19]), while the third anagram is virtually impossible to solve (solved by ca. 1% [19]). In the second part of the anagram task, respondents are presented with the solutions and are given the opportunity to participate in a lottery for 100€, 50€ and 30€ under the condition that they were able to solve all three anagrams. Respondents are asked whether they were able to solve all three anagrams in time. The two available answer options are: “No, I solved fewer than three anagrams” and “Yes, I solved all three anagrams (opportunity to participate in the lottery at the end of the survey)”. These answer options are explicitly designed to motivate respondents to overreport their performance. Due to the indirect query of the number of solved anagrams, respondents should feel safe that they will not be exposed as cheaters. However, because solving all three anagrams is virtually impossible, all respondents claiming to have found all solutions are categorized as cheaters.

#### Sensitive question

The sensitive question read: “On the anagram task, I claimed that I had solved more anagrams than I had actually solved”. It was asked in either the CWM detailed, CWM short or DQ format (between-subjects). In the DQ format, respondents simply had to indicate whether the sensitive question was “true” or “false”. In the CWM format, respondents had to answer two statements simultaneously: the aforementioned sensitive statement and a non-sensitive statement with known prevalence *p*: “I was born in November or December” (*p* = .158 according to official birth statistics [36]). The answer options read: “Both statements are true or both statements are false” versus “exactly one statement is true (irrespective of which one)”. Respondents in the CWM brief condition received brief instructions on how to answer the question, and were informed that the response format would protect their privacy as their birth month would remain unknown to the researchers. In addition to the instructions provided in the CWM brief condition, respondents in the CWM detailed condition were further informed that the researchers would use the relative probability of being born in November or December to compute the share of people who agreed to the sensitive statement on the sample level, but that their individual answers would remain confidential. Moreover, respondents were provided with four comprehension questions capturing whether they had understood how to answer the CWM question. As an example, the first comprehension question was as follows:

“Assuming you were born in February, and assuming you had *not* exaggerated on your report of the number of solved anagrams. Which answer would you have to give?”

The answer options read: “I would have to answer ‘both statements are true or both statements are false’” and “I would have to answer ‘exactly one statement is true (irrespective of which one)’”, and were presented in randomized order. The four comprehension questions covered all four combinations of respondents potentially holding or not holding the sensitive attribute (exaggerating their report of the number of solved anagrams) as well as the non-sensitive attribute used for randomization (being born in November or December). On the subsequent page, respondents received feedback on their responses. If the respondents failed to provide correct responses to any of the comprehension questions, the detailed instructions and those comprehension questions that were not answered correctly were repeated up to two times. The presentation ended when respondents had provided correct responses to all four comprehension questions, or when they failed to provide a correct response to at least one of the questions three times. Subsequently, respondents were presented with two additional questions capturing whether they had understood how the CWM protected their privacy. The first of these questions read:

“Imagine you had chosen the option ‘Both statements are true or both statements are false’. What could someone who does not know your birth month infer from your choice?”

The answer options read: “He could infer that you had exaggerated your report of the number of solved anagrams”, “He could infer that you reported the number of solved anagrams truthfully”, and “He could infer neither of the above”, presented in fixed order. The second question covered the case in which respondents had hypothetically chosen the second answer option with respect to the sensitive statement (“exactly one of the statements is true (irrespective of which one)”). Again, respondents received feedback on their answers, and the detailed instructions and questions were repeatedly presented up to two times if not solved correctly. After the comprehension questions, the respondents were presented with the actual sensitive question. While the detailed instructions on the questioning technique were still available, respondents were explicitly told that they should now choose the response that aligns with their own personal behavior, and that their birth month would remain unknown to the researchers.

#### Self-reported response behavior and perception of the questioning technique

Respondents were asked to evaluate the sensitive question by indicating how strongly they agreed with several statements. These statements read: “The question was comprehensible” (perceived comprehensibility), “The question guaranteed the confidentiality of my response” (perceived confidentiality), “I clearly knew which answer to pick” (perceived clarity), and “I just ticked anything” (random response; this variable was then reverse-coded, with higher values indicating less random responding). All statements were rated on a 7-point Likert-type scale ranging from 1 (*strongly disagree*) to 7 (*strongly agree*).

### Procedure

Respondents filled in an online questionnaire that began with a short introduction, followed by demographic questions asking about their gender, age, native language and highest school-leaving qualification. They were then given the instructions for the anagram task and had the opportunity to practice the task with two example anagrams. Next, respondents were informed that the actual task would now start and that if they could solve all three anagrams, they could take part in a lottery for 100€, 50€ and 30€. After the anagram task, they were given the opportunity to cheat on reporting the number of solved anagrams as described above. Subsequently, they were queried with regard to their cheating behavior in the anagram task in either the DQ, the CWM detailed, or the CWM brief format (between-subjects). After the sensitive question, the respondents were asked to evaluate the questioning technique, were debriefed and were then given the opportunity to participate in the lottery. In order to avoid discriminating against honest respondents, all respondents were given the opportunity to participate in the lottery regardless of whether they had answered honestly or dishonestly.

### Statistical analyses

For parameter estimation and comparison, we formulated multinomial processing tree (MPT) models [37, 38] following the procedure outlined in previous studies [35, 39, 40]. The parameter π represents the prevalence of the sensitive attribute (cheating on the anagram task) and the parameter *p* represents the known prevalence of the non-sensitive attribute used for randomization (birth month, *p* = .158 according to official birth statistics [36]). Maximum likelihood estimates were obtained using the expectation maximization algorithm [41, 42] implemented in the software multiTree [43], version 0.46. Parameter comparisons and restrictions were assessed via differences in the asymptotically χ²-distributed log-likelihood statistic *G*² between an unrestricted baseline model and a restricted alternative model (e.g. π_CWM_detailed_ = π_DQ_ or π_CWM_detailed_ = .00).

To more thoroughly investigate the validity of the obtained estimates, we transferred the approach of analyzing false positives and false negatives detailed in Höglinger and Jann [22] to the multinomial framework. To this end, we first split the sample into two parts: respondents who claimed to have solved all three anagrams in the anagram task were categorized as cheaters, while respondents who reported having solved fewer than three anagrams were considered honest respondents. This categorization is justified by the fact that solving all three anagrams has been shown to be virtually impossible in a previous study [19]. We then formulated separate multinomial processing trees for cheaters and honest respondents, and within these subsamples, for the DQ and CWM conditions. Hence, the false positive rate was estimated as the proportion of carriers of the sensitive attribute (π) within the sub-sample of honest respondents for the respective questioning technique, and the false negative rate was estimated as the proportion of non-carriers (1- π) in the sub-sample of cheaters.

## Results

### Parameter estimates, false positives and false negatives

Our analyses revealed significantly higher prevalence estimates in both CWM conditions (detailed: $\hat{\pi }$ = 25.48%, *SE* = 2.21%; brief: $\hat{\pi }$ = 30.78%, *SE* = 2.07%) as compared to the DQ condition ($\hat{\pi }$ = 11.79%, *SE* = 1.34%); CWM detailed vs. DQ: Δ$\hat{\pi }$ = 13.69%, Δ*G*²(1) = 27.94, *p* < .001; CWM brief vs. DQ: Δ$\hat{\pi }$ = 18.99%, Δ*G*²(1) = 56.74, *p* < .001; CWM detailed vs. brief: Δ$\hat{\pi }$ = 5.30%, Δ*G*²(1) = 3.06, *p* = .080. The known prevalence of the sensitive attribute (DQ: 58.93%, CWM brief: 56.70%, CWM detailed: 59.26%) did not differ across conditions, χ²(2) = 1.63, *p* = .442, *Cramer’s V* = .03, and was underestimated by all questioning techniques; CWM detailed vs. known prevalence: Δ$\hat{\pi }$ = 33.78%, Δ*G*²(1) = 210.75, *p* < .001; CWM brief vs. known prevalence: Δ$\hat{\pi }$ = 25.92%, Δ*G*²(1) = 147.47, *p* < .001; DQ vs. known prevalence: Δ$\hat{\pi }$ = 47.14%, Δ*G*²(1) = 558.69, *p* < .001. Thus, the CWM met a weak (“more is better”), but not a strong validation criterion, as it still substantially underestimated the known prevalence. Moreover, we detected substantial rates of false positives in all experimental groups (see Table 1 and S1 Appendix), with the highest rates in both CWM conditions (CWM detailed: 13.08%, CWM brief: 14.32%, DQ: 2.53%; this corresponds to a specificity of CWM detailed: 86.92%, CWM brief: 85.68%, DQ: 97.47%). In all conditions, the false positive rates were significantly higher than 0%, CWM detailed vs. 0%: Δ$\hat{\pi }$ = 13.08%, Δ*G*²(1) = 20.99, *p* < .001, CWM brief vs. 0%: Δ$\hat{\pi }$ = 14.32%, Δ*G*²(1) = 31.70, *p* < .001, DQ vs. 0%: Δ$\hat{\pi }$ = 2.53%, Δ*G*²(1) = 165.09, *p* < .001. The false positive rates in both CWM conditions did not significantly differ from each other, CWM detailed vs. CWM brief: Δ$\hat{\pi }$ = 1.24%, Δ*G*²(1) = 0.08, *p* = .771, but they were significantly higher than the false positive rate in the DQ condition, CWM detailed vs. DQ: Δ$\hat{\pi }$ = 10.55%, Δ*G*²(1) = 10.82, *p* = .001, CWM brief vs. DQ: Δ$\hat{\pi }$ = 11.79%, Δ*G*²(1) = 15.86, *p* < .001. Comparatively high rates of false negatives were observed in all conditions (see Table 1 and S2 Appendix). While the highest rate was found in the DQ condition (81.77%; sensitivity: 18.23%), the false negative was also substantial in both CWM conditions (detailed: 65.99%, sensitivity: 34.01%; brief: 56.65%; sensitivity: 43.35%). In all conditions, the false negative rates were significantly higher than 0%, CWM detailed vs. 0%: Δ$\hat{\pi }$ = 65.99%, Δ*G*²(1) = 601.97, *p* < .001, CWM brief vs. 0%: Δ$\hat{\pi }$ = 56.65%, Δ*G*²(1) = 522.21, *p* < .001, DQ vs. 0%: Δ$\hat{\pi }$ = 81.77%, Δ*G*²(1) = 9918.94, *p* < .001. The false negative rates in all conditions differed significantly from each other, CWM detailed vs. CWM brief: Δ$\hat{\pi }$ = 9.34%, Δ*G*²(1) = 5.15, *p* = .023, CWM detailed vs. DQ: Δ$\hat{\pi }$ = 15.78%, Δ*G*²(1) = 18.47, *p* = .001, CWM brief vs. DQ: Δ$\hat{\pi }$ = 25.12%, Δ*G*²(1) = 47.71, *p* = .001.

**Table 1:** False positives and false negatives in the total sample and split by randomness of responses, perceived comprehensibility, perceived confidentiality and perceived clarity of the questioning technique (standard errors in parentheses).

	False Positives (in %)			False Negatives (in %)		
	DQ	CWM brief	CWM detailed	DQ	CWM brief	CWM detailed
Total sample (*N* = 2713)	2.53 (1.02)	14.32 (2.84)	13.08 (3.17)	81.77 (2.09)	56.65 (2.83)	65.99 (2.97)
Randomness of responses						
non-random (*N* = 2194)	1.40 (0.80)	13.69 (2.98)	6.14 (3.35)	84.97 (2.11)	57.76 (3.14)	70.94 (3.51)
random (*N* = 519)	13.64 (7.32)	19.90 (9.33)	36.34 (7.53)	64.82 (6.50)	51.78 (6.59)	54.93 (5.47)
Perceived comprehensibility						
comprehensible (*N* = 1244)	1.10 (0.77)	15.89 (3.94)	8.83 (6.48)	85.20 (2.25)	58.17 (3.89)	72.07 (6.77)
incomprehensible (*N* = 1469)	7.27 (3.50)	12.51 (4.10)	14.28 (3.63)	72.22 (4.72)	54.94 (4.14)	64.62 (3.30)
Perceived confidentiality						
confidential (*N* = 1163)	1.42 (1.00)	15.97 (4.25)	6.36 (5.07)	87.03 (2.47)	55.70 (4.24)	70.77 (5.28)
not confidential (*N* = 1550)	4.17 (2.04)	12.91 (3.82)	16.52 (4.02)	75.48 (3.46)	57.41 (3.81)	63.89 (3.59)
Perceived clarity						
clear (*N* = 1551)	1.14 (0.80)	14.35 (3.33)	7.40 (5.04)	85.06 (2.21)	53.96 (3.54)	67.48 (5.23)
unclear (*N* = 1162)	6.45 (3.12)	14.25 (5.45)	16.15 (4.04)	70.89 (5.11)	61.51 (4.71)	65.29 (3.61)

DQ = direct questioning, CWM brief = crosswise model with brief instructions, CWM detailed = crosswise model with detailed instructions and comprehension questions.

#### Effects of education

**Fig 2:**
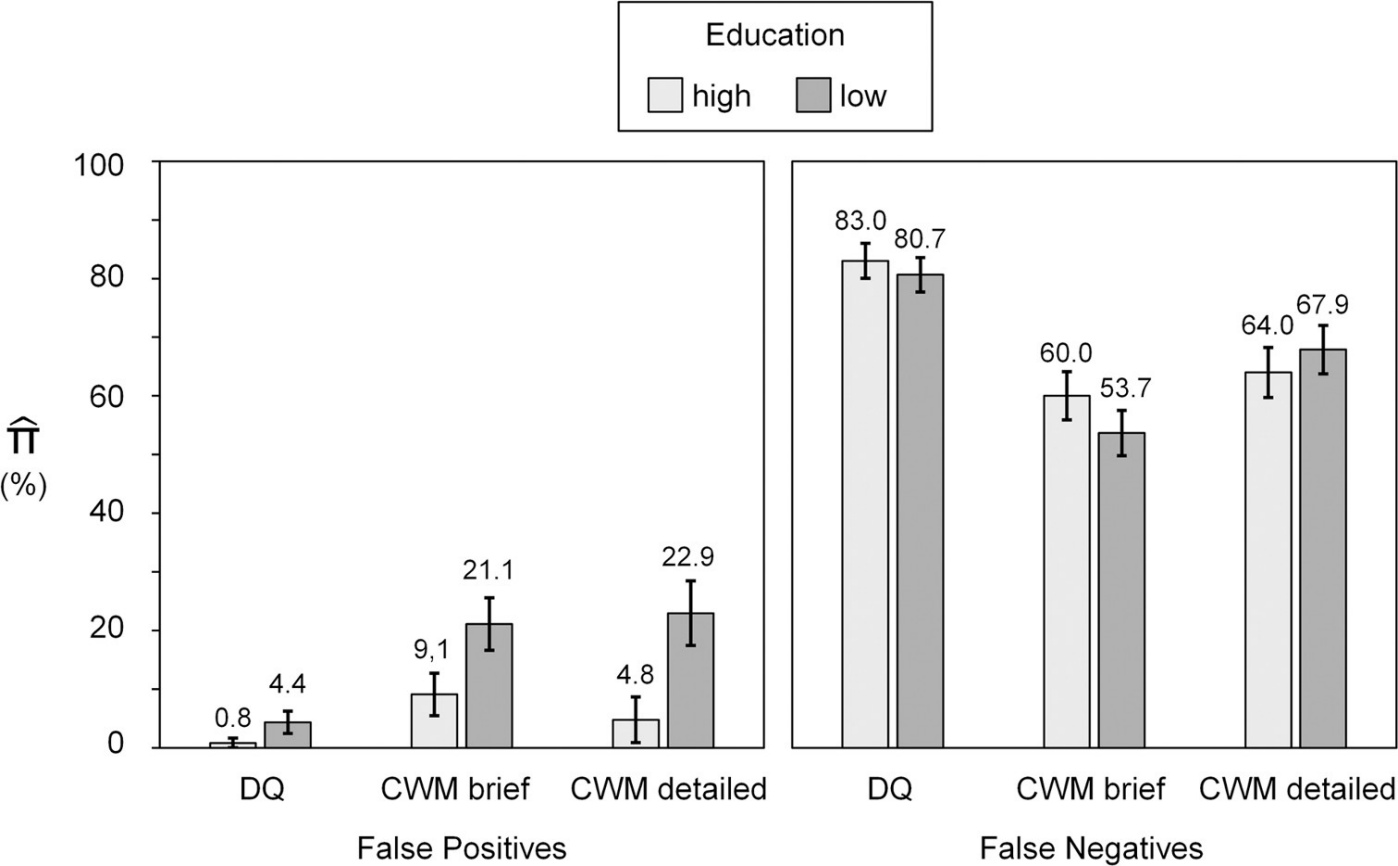
False positives and false negatives as a function of level of education and condition. DQ = direct questioning, CWM brief = crosswise model with brief instructions, CWM detailed = crosswise model with detailed instructions and comprehension questions.

A split by level of education (high vs. low) revealed that false positives were particularly prevalent among lower-educated respondents (see Fig 2). In both CWM conditions, false positive rates were significantly lower for higher-educated respondents than for lower-educated respondents, CWM brief: Δ$\hat{\pi }$ = 11.91%, Δ*G*²(1) = 4.33, *p* = .038; CWM detailed: Δ$\hat{\pi }$ = 18.16%, Δ*G*²(1) = 8.13, *p* = .004. In the DQ condition, this tendency was not significant, Δ$\hat{\pi }$ = 3.53%, Δ*G*²(1) = 3.23, *p* = .072. False negative rates (see Fig 1) were not affected by level of education, DQ: Δ$\hat{\pi }$ = 2.36%, Δ*G²*(1) = 0.32, *p* < .574; CWM brief: Δ$\hat{\pi }$ = 6.98%, Δ*G*²(1) = 1.51, *p* = .220; CWM detailed: Δ$\hat{\pi }$ = 3.90%, Δ*G*²(1) = 0.43, *p* = .511.

#### Selection by comprehension questions

To more thoroughly evaluate respondents’ objective comprehension of the CWM instructions, we analyzed the rates of correct responses to the comprehension questions in the CWM detailed condition. Comprehension Questions 1 to 6 were passed by a total of 68.83% of respondents; 12.45% provided correct responses to all comprehension questions in the first attempt, 56.38% in the second or third attempt. Higher-educated respondents were more likely to provide correct answers to all comprehension questions in the first attempt (18.71%) as well as in the second or third attempt (59.76%) than lower-educated respondents (first attempt: 5.89%, second or third attempt: 52.84%), χ²(2) = 64.46, *p* < .001, *Cramer’s V* = .26.

To determine whether comprehension questions can be used to improve overall data quality, we exploratively repeated the analyses of false positives and false negatives in the CWM detailed condition including only those respondents who were eventually able to correctly answer all comprehension questions (hereinafter referred to as *respondents with high understanding*, *N* = 669, 68.8% of respondents in the CWM detailed condition). For higher-educated respondents, the false positive rate dropped from 4.78% (*SE* = 3.92%) when including all respondents in the CWM detailed condition to 0.00% (*SE* = 4.35%) in the subgroup of respondents with high understanding. For lower-educated respondents, however, the false positive rate slightly increased from 22.94% (*SE* = 5.05%) when including all respondents in the CWM detailed condition to 25.17% (*SE* = 6.68%) when considering only the subgroup of respondents with high understanding. Moreover, among the subgroup of respondents with high understanding, the false positive rate was significantly lower for higher-educated respondents compared to lower-educated respondents, Δ$\hat{\pi }$ = 25.17%, Δ*G*²(1) = 14.15, *p* < .001. In both educational groups, the false negative rate was higher in the subsample of respondents with high understanding compared to an analysis without sample constraints (higher education: all respondents in the CWM detailed condition: 64.00%, *SE* = 4.27%, respondents with high understanding: 70.79%, *SE* = 4.75%; low education: all respondents in the CWM detailed condition: 67.90%, *SE* = 4.13%, respondents with high understanding: 73.24%, *SE* = 5.27%). Within the subgroup of respondents in the CWM detailed condition with high understanding, false negative rates did not differ with regard to education, Δ$\hat{\pi }$ = 2.36%, Δ*G*²(1) = 0.12, *p* = .730.

Overall, these results suggest that false positives can be effectively reduced by comprehension questions when the instructions are detailed, but only among higher-educated samples. Moreover, this comes at the expense of an increase in false negatives.

#### Effects of self-reported response behavior and perception of the questioning technique

Table 2 reports descriptive statistics for self-ratings of randomness of responses, perceived comprehensibility, perceived confidentiality, and perceived clarity of the questioning techniques. All of these variables were significantly intercorrelated (see Table 3); a Cronbach’s alpha of .70 indicated that they measured a homogeneous construct. ANOVAs and Bonferroni-corrected post-hoc tests indicated that the CWM detailed condition was evaluated as less understandable, less confidential and less clear than the CWM brief condition, which in turn was evaluated as worse than the DQ condition on all of these variables. Moreover, respondents in the CWM detailed condition indicated significantly more random responses than respondents in the CWM brief or DQ conditions (see Table 2).

**Table 2:** Descriptive statistics and results of ANOVAs for self-reported response behaviors and perceptions of the questioning technique split by condition.

	CWM detailed	CWM brief	DQ	F (2,2703)	*p*	η_p_
	*M (SD)*	*M (SD)*	*M (SD)*			
Randomness of responses	6.29*	6.54	6.60	13.44	< .001	.01
	(1.41)	(1.30)	(1.23)			
Perceived comprehensibility	4.47*	6.01*	6.53*	409.99	< .001	.23
	(1.87)	(1.43)	(1.04)			
Perceived confidentiality	5.27*	5.70*	6.07*	49.69	< .001	.04
	(1.64)	(1.58)	(1.39)			
Perceived clarity	4.55*	5.82*	6.12*	249.92	< .001	.16
	(1.75)	(1.48)	(1.33)			

All variables were assessed on a 7-point Likert-type scale with higher values indicating more favorable evaluations. Randomness of responses was originally reverse-coded, but was inverted to facilitate the interpretability of means.

* Bonferroni-corrected post-hoc tests revealed that these conditions significantly differed from all other conditions (all *p* < .001).

**Table 3:** Correlations between self-reported response behaviors and perceptions of the questioning technique.

	Randomness of responses	Perceived comprehensibility	Perceived confidentiality	Perceived clarity
Randomness of responses	-	.21*	.15*	.17*
Perceived comprehensibility		-	.50*	.64*
Perceived confidentiality			-	.42*
Perceived clarity				-

All variables were assessed on a 7-point Likert-type scale with higher values indicating more favorable evaluations. Randomness of responses was originally reverse-coded, but was inverted to facilitate the interpretability of means.

* *p* < .001

Spearman rank correlations revealed that respondents who performed better on the comprehension questions (1 = ‘failed at least one comprehension question in the third attempt’, 2 = ‘comprehension questions solved in second or third attempt’, 3 = ‘comprehension questions solved in first attempt’), indicated lower rates of random responses (r_*s*_ = -.31, *p* < .001, *n* = 972) as well as higher perceived comprehensibility (r_*s*_ = .30, *p* < .001, *n* = 972), confidentiality (r_*s*_ = .21, *p* < .001, *n* = 972) and subjective clarity of the questioning technique (r_*s*_ = .35, *p* < .001, *n* = 972).

To determine whether respondents’ self-assessment of the randomness of their responses was associated with the validity of the results obtained, we identified respondents who had indicated that they *strongly disagreed* with the statement “I simply ticked anything” (80.9% of the sample). These respondents were classified as having provided “non-random responses”, while all other respondents were considered to have provided “random responses”. An exploratory split by this moderator variable revealed that false positive rates were substantially lower among respondents who indicated having provided non-random responses; this pattern was observed in both the CWM detailed and the DQ conditions, but not in the CWM brief condition. However, this decrease in false positive rates came at the expense of an increase in false negative rates in both the CWM detailed and the DQ condition. Similar results were observed for exploratory median splits of perceived comprehensibility, perceived confidentiality and perceived clarity of the questioning technique: Higher values on these variables were associated with reduced false positives, but also increased false negatives in the CWM detailed and the DQ conditions. However, these tendencies were only significant in the DQ condition, and only for splits with reference to perceived comprehensibility and perceived clarity. In a multinomial modeling framework based on binary trees, continuous variables such as perceived comprehensibility or perceived confidentiality cannot be included directly. To transform these variables in a binary format, we therefore applied median splits. For detailed statistics on these analyses, see Table 1 and S1 and S2 Appendices.

### Completion times

A Kruskal-Wallis test showed that completion times for the experimental section of the questionnaire differed significantly across the three experimental conditions, χ²(2) = 2232.53, *p* < .001. Dunn-Bonferroni corrected post-hoc tests revealed that the detailed CWM instructions were associated with higher completion times (median: 380 seconds) than the brief CWM instructions (median: 43 seconds), and the brief instructions with higher completion times than the DQ instructions (median: 9 seconds), DQ vs. CWM brief: *z* = 20.14, *p* < .001; DQ vs CWM detailed: *z* = 45.61, *p* < .001; CWM brief vs. CWM detailed: *z* = 31.56, *p* < .001.

## Discussion

In the present study, we investigated an apparent contradiction in the scientific literature regarding the validity of the crosswise model (CWM [8]), an indirect questioning technique designed to control for socially desirable responding. While numerous studies suggest that prevalence estimates obtained via the CWM are highly valid [12–14, 18, 19], recent work by Höglinger and Diekmann [21] and Höglinger and Jann [22] suggests that the model tends to produce false positives in certain situations. Building upon these findings, we sought to identify conditions under which false positives occur in applications of the CWM and investigated what measures can be taken to effectively reduce the false positive rate to a minimum. The core idea was that false positives might be caused by an insufficient understanding of the instructions. To test this idea, we conducted a strong validation and compared the validity of estimates obtained via conventional direct questions (DQ) with the validity of estimates obtained via the CWM in two groups, one of which received only brief instructions on how to answer the sensitive question (CWM brief), and the other of which received more detailed information on the procedure and had to answer several comprehension questions (CWM detailed).

Overall, the CWM led to significantly higher prevalence estimates than DQ, thus meeting the “more is better” criterion on the aggregate level. However, both DQ and the CWM severely underestimated the known prevalence of the sensitive attribute, thus failing a strong validation. Moreover, in line with our hypotheses, we found higher rates of false positives for both CWM conditions as compared to the DQ condition. In contrast, false negatives were significantly more common in the DQ condition as compared to both CWM conditions. The hypotheses that false positives occur less frequently in CWM applications when respondents have a deep understanding of the method (detailed CWM) compared to a superficial understanding (CWM brief), and that false positives occur more frequently in lower-educated than in higher-educated respondents, were only partially confirmed. As expected, detailed instructions combined with comprehension questions led to lower rates of false positives, but only within the subgroup of higher-educated respondents. However, neither detailed instructions and comprehension questions nor higher education completely eliminated false positives in the CWM at the individual level.

The results of our study generally support the findings of Höglinger and Diekmann [21] and Höglinger and Jann [22] that the CWM in its original form tends to produce false positives. However, in contrast to previous studies that did not experimentally investigate potential moderators of the false positive rate [21, 22], the present study showed that satisfactorily low rates of false positives could be achieved by the use of extensive instructions in combination with comprehension questions for one subgroup of respondents. Specifically, low false positive rates were observed in the sub-sample of higher-educated respondents and among participants who indicated that they did not provide random answers and who perceived the questions as easily comprehensible and as protecting their confidentiality. False positives were completely eliminated (0.0%) among the higher-educated respondents who passed all comprehension questions. The positive association between education level and CWM performance is consistent with the results of a recent study showing that higher-educated respondents are better at understanding CWM instructions [20]. However, in the present study, the beneficial effect of comprehension checks on the validity of prevalence estimates came at the expense of higher dropout rates and higher completion times. The fact that CWM estimates are not equally valid for respondents with different levels of education furthermore implies that correlations between sensitive attributes and covariates such as education are likely biased when using the CWM.

Interestingly, in the current study, substantial rates of false positives were also observed in the DQ condition. This observation is striking given that DQ does not include complex instructions, but only requires respondents to make a rather simple decision of agreeing or disagreeing with a statement. Hence, this finding seems to indicate that the issue of false positives is not a specific drawback of indirect questioning techniques such as the CWM, but extends to situations in which prevalence estimates are based on self-reports of any kind. In line with this, Hoffmann et al. [20] found that the rate of incorrect answers in a DQ condition was about 10%. In another study by Bishop et al. [44], a substantial number of respondents took a clear stance on a purely fictional issue, which impressively illustrates that self-reports must be interpreted cautiously. Such response patterns may be due to careless responding, straightlining, or non-serious participation, which are common phenomena in self-reports and have been shown to impair data quality [45–49]. These concepts are closely related to the ‘randomness’ of responses in the present study. The finding that the false positive rate was lower among respondents who indicated having provided non-random responses than among respondents who indicated having provided random responses lends further support to the assumption that false positives are a product of careless responding and non-serious participation.

One point that has received little attention in the recently published literature reporting strong validations of indirect questioning techniques is the fact that some studies have also found very high rates of false negatives. False negatives refer to the proportion of carriers of a sensitive attribute that are incorrectly categorized as being non-carriers. While false positives can lead to an undesired overestimation, false negatives carry the risk of underestimating the prevalence of sensitive attributes. It was precisely to avoid this problem that indirect questioning techniques were introduced in the first place. In our study, significantly lower rates of false negatives were observed in both CWM conditions compared to the DQ condition. This finding provides clear evidence of an advantage of CWM questions over conventional direct questions, namely a higher proportion of correctly identified carriers of the sensitive attribute. Remarkably, for the CWM, the rate of false negatives was considerably higher than the rate of false positives. Moreover, the rates of false positives and false negatives were interdependent: A reduction in false positives (e.g. by selecting only those respondents who passed the comprehension checks) led to an increase in false negatives, presumably due to the application of a more conservative criterion.

Overall, with regard to the prevalence estimates obtained, the deflating influence of false negatives clearly outweighed the inflating influence of false positives. This led to a severe underestimation of the known prevalence of the sensitive attribute in all conditions. It seems likely that the relative effects of false positives and false negatives depend on the true prevalence of the sensitive item under study. In the present study, as well as in many other studies (e.g. [13, 24, 40, 50, 51]). the prevalence of the sensitive attribute was relatively high, and substantially higher than 0%. If, however, attributes with a true prevalence of approximately 0% are investigated, almost the entire sample consists of non-carriers. Therefore, only false positives, but no false negatives, can be observed. In this special case, any false positive rate higher than 0% will necessarily lead to an overestimation of the true prevalence [21]. Moreover, the present study showed that the rate of false positives and false negatives differs between different samples. Against the background of meta-analytical data [5], it seems reasonable to assume that overall, the effect of false negatives outweighs the effect of false positives and RRTs thus more likely under- rather than overestimated the true prevalence in previous studies. In light of this, prevalence estimates for sensitive personal attributes with a prevalence substantially higher than 0% that were obtained in previous studies using the CWM (e.g. xenophobia [10]; prejudice against female leaders [18]; plagiarism [12]) or other indirect questioning techniques (e.g. doping [52]) were most likely underestimations rather than overestimates of the population values.

In summary, our results are in line with the findings of two meta-analyses on RRT studies [5]: Prevalence estimates for sensitive attributes obtained via indirect questioning techniques such as the CWM demonstrably underestimate the true value due to substantial rates of false negatives; nevertheless, CWM estimates seem superior to estimates obtained via a conventional DQ approach—at least for sensitive attributes with a prevalence substantially higher than 0%—as they more closely reflect the ground truth.

### Limitations and future research directions

While the current study will hopefully contribute to a better understanding of the conditions under which false positives and false negatives occur in applications of the CWM, some questions cannot be answered on the basis of our data and should therefore be addressed in future research.

First, it would be interesting to gain a deeper understanding of the cognitive processes involved in the formation of false positives and false negatives in the CWM. As corroborated by our data, false positives are most likely a product of inadvertent instruction non-adherence. It seems rather unlikely that non-carriers try to make themselves appear to be carriers of the sensitive attribute by deliberately choosing an answer that does not correspond to their actual status. False negatives, however, could be a mixture of carriers inadvertently providing a false response due to instruction miscomprehension, and carriers deliberately choosing the answer that minimizes the probability of them being identified as a carrier. While a particular advantage of the CWM is that none of the answer options clearly excludes the possibility of carrying the sensitive attribute, one of the answer options is still associated with a lower risk of being identified as a carrier, depending on the randomization probability *p*. Given our data, we cannot answer whether and to what extent carriers pursued this strategy. Hence, future research should address this question via methods such as personal interviews and open-ended questions about how respondents arrived at their specific answers.

Second, our data cannot uncover the processes responsible for the large share of inaccurate responses provided by lower-educated respondents. While false positives could be reduced among higher-educated respondents when detailed instructions and comprehension questions were included, the false positive rate among lower-educated respondents was not affected by such measures. Future research projects should therefore continue to optimize conditions until both higher- and lower-educated respondents are willing and able to provide accurate responses. This is of particular importance when the attribute under investigation is moderated by respondents’ level of education (e.g., negative attitudes towards foreigners [29]), because differential comprehension levels might lead to erroneous conclusions.

Third, the present study highlights that respondents’ thorough comprehension of indirect questions is a necessary prerequisite for obtaining valid results. For this reason, the exact implementation of the questioning technique seems crucial. However, implementation details are often unknown due to insufficient documentation, and a considerable amount of research focuses exclusively on the development of new statistical models and ignores questions of feasibility and implementation. We therefore recommend that future research focus more on the procedural implementation and comprehensibility of indirect questioning techniques. In addition, we encourage researchers to contribute to the improvement of tools that capture respondents’ understanding of indirect questions, such as the comprehension checks employed in the present study. It would be desirable to design these measures in a way that ensures a thorough comprehension of indirect questioning techniques even among lower-educated samples.

Fourth, it remains unclear why the CWM was perceived as less confidential overall than DQ. This finding contrasts with the objective confidentiality guaranteed by the CWM and also contradicts a recent study in which the CWM’s subjective privacy protection was rated significantly higher than the protection provided by DQ [20]. Possible reasons might include the perceived high complexity of the CWM instructions as well as the between-subjects design of the current study, which could have prevented the respondents from establishing common reference frames [cf. 53]. Moreover, it is unclear why the CWM detailed format, which was supposed to enhance understanding, was perceived as less comprehensible than the CWM brief format, and why respondents were less sure of what to do in the CWM detailed than in the CWM brief condition. It seems likely that the comprehension questions in the CWM detailed condition raised respondents’ awareness of the complexity of the CWM instructions, leading them to subjectively perceive the questioning format as rather complicated, whereas respondents in the CWM brief condition received no feedback on their understanding of the instructions and thus might not have realized that they did not understand the procedure properly. Once again, the between-subjects design of the current study may have also prevented respondents from establishing common reference frames [cf. 53].

Finally, the harmful influence of false negatives was substantially more pronounced in the present study than the influence of false positives. Overall, this led to an underestimation of the known prevalence. Future research should thus also try to identify conditions under which false negatives can be avoided. To this end, we recommend that studies employing a strong validation approach, comprehensive instructions and comprehension checks also be conducted for other indirect questioning techniques (e.g. cheating detection models). Such studies should ideally compare the validity of different models across sensitive attributes with varying prevalence in order to explore the potential influence of the population value on the validity of the prevalence estimates obtained.

### Practical implications

In light of the current results, the CWM can be recommended for application if–and only if–the sample under investigation is highly educated, and detailed instructions and comprehension questions are used. As the present results also show, however, the desirable positive effect of detailed instructions and comprehension questions on the validity of the prevalence estimates obtained comes at the expense of higher dropout rates and higher completion times. Moreover, our results call into question the application of the CWM in its current format among lower-educated samples. In order to obtain valid answers among lower-educated respondents, more research seems needed to find ways of improving such respondents’ instruction comprehension. The present results also highlight the importance of including measures of instruction comprehension as well as specific aspects of respondents’ subjective experience (such as perceived confidentiality and randomness of responses) in surveys of sensitive personal attributes. Moreover, the present study underscores the importance of strong validations, since only individual-level data allow for the detection of false positives and false negatives, and thus for a comprehensive assessment of a method’s validity [6, 22].

## Conclusion

The present study confirmed the assumption that the CWM tends to produce false positives. It also showed that the problem of false positives is not specific to indirect questioning techniques, but rather seems to be a drawback of self-reports of any kind, including conventional DQ. On the aggregate level, there were many more false negatives than false positives, resulting in severe underestimations of the prevalence of the sensitive attribute across all questioning techniques. However, taking both false positives and false negatives into account, the CWM clearly outperformed DQ in terms of aggregate validity. Our findings therefore further suggest that CWM estimates in previous studies of sensitive attributes with a prevalence substantially higher than 0% were more likely to be underestimates rather than overestimates of the true prevalence of sensitive attributes.

## Supporting information

S1 Appendix Parameter comparisons of false positives. (PDF)

S2 Appendix Parameter comparisons of false negatives. (PDF)

S1 File Multi tree equations. MultiTree equations for the estimation of π, false positives and false negatives in a multinomial model.
(PDF)

S2 File Study materials. Original instructions for the sensitive question by experimental condition.
(PDF)

S1 Data Empirically observed answer frequencies for the attributes used for parameter estimation in multiTree. (PDF)
