# Supplementary material for: Can detailed instructions and comprehension checks increase the validity of crosswise model estimates?
Source: PLoS One. 2020 Jun 30;15(6):e0235403. doi: 10.1371/journal.pone.0235403 (PMC7326177; doi:10.1371/journal.pone.0235403)
Supplement: S1 Appendix — (PDF) [file pone.0235403.s001.pdf]

Supporting Information File: Appendix

Parameter comparisons of false positives for the total sample and split by randomness of responses, perceived comprehensibility, perceived confidentiality and perceived clarity of the questioning technique

Parameter Comparisons: False Positives							
						Model fit	
			Parameter 1 (in %)	Parameter 2 (in %)	|*Difference*| (in %)	ΔG^2 (df = 1)	p
Total Sample							
		$FP_{CWM\ detailed} = FP_{CWM\ brief}$	13.08	14.32	1.24	0.08	= .771
		$FP_{CWM\ detailed} = FP_{DQ}$	13.08	2.53	10.55	10.82	= .001*
		$FP_{CWM\ brief} = FP_{DQ}$	14.32	2.53	11.79	15.86	< .001*
		$FP_{CWM\ detailed}$ = 0%	13.08	0.00	13.08	20.99	< .001*
		$FP_{CWM\ brief}$ = 0%	14.32	0.00	14.32	31.70	< .001*
		$FP_{CWM\ brief}$ = 0%	2.53	0.00	2.53	165.09	< .001*
Randomness of responses							
	Non-random	$FP_{CWM\ detailed} = FP_{CWM\ brief}$	6.14	13.69	7.55	2.77	= .096
		$FP_{CWM\ detailed} = FP_{DQ}$	6.14	1.40	4.74	2.03	= .154
		$FP_{CWM\ brief} = FP_{DQ}$	13.69	1.40	12.29	17.17	< .001*
		$FP_{CWM\ detailed}$ = 0%	6.14	0.00	6.14	3.79	= .052
		$FP_{CWM\ brief}$ = 0%	13.69	0.00	13.69	26.18	< .001*
		$FP_{CWM\ brief}$ = 0%	1.40	0.00	1.40	78.93	< .001*
	Random	$FP_{CWM\ detailed} = FP_{CWM\ brief}$	36.34	19.90	16.44	1.81	= .178
		$FP_{CWM\ detailed} = FP_{DQ}$	36.34	13.64	22.70	3.87	= .049*
		$FP_{CWM\ brief} = FP_{DQ}$	19.90	13.64	6.26	0.27	= .601
		$FP_{CWM\ detailed}$ = 0%	36.34	0.00	36.34	32.16	< .001*
		$FP_{CWM\ brief}$ = 0%	19.90	0.00	19.90	5.95	= .015*
		$FP_{CWM\ brief}$ = 0%	13.64	0.00	13.64	93.00	< .001*

CWM detailed	$FP_{non\text{-}random} = FP_{random}$	6.14	36.34	30.2	14.96	< .001*
CWM brief	$FP_{non\text{-}random} = FP_{random}$	13.69	19.90	6.21	0.42	= .516
DQ	$FP_{non\text{-}random} = FP_{random}$	1.40	13.64	12.24	6.85	= .009*
Perceived comprehensibility						
Comprehensible	$FP_{CWM\ detailed} = FP_{CWM\ brief}$	8.83	16.03	7.20	0.83	= .363
	$FP_{CWM\ detailed} = FP_{DQ}$	8.83	1.10	7.73	1.70	= .192
	$FP_{CWM\ brief} = FP_{DQ}$	15.89	1.10	14.79	15.79	< .001*
	$FP_{CWM\ detailed} = 0\%$	8.83	0.00	8.83	2.18	= .140
	$FP_{CWM\ brief} = 0\%$	15.89	0.00	15.89	20.65	< .001*
	$FP_{CWM\ brief} = 0\%$	1.10	0.00	1.10	51.66	< .001*
Incomprehensible	$FP_{CWM\ detailed} = FP_{CWM\ brief}$	14.28	12.51	1.77	0.10	= .748
	$FP_{CWM\ detailed} = FP_{DQ}$	14.28	7.27	7.01	1.61	= .205
	$FP_{CWM\ brief} = FP_{DQ}$	12.51	7.27	5.24	0.88	= .347
	$FP_{CWM\ detailed} = 0\%$	14.28	0.00	14.28	19.33	< .001*
	$FP_{CWM\ brief} = 0\%$	12.51	0.00	12.51	11.40	< .001*
	$FP_{CWM\ brief} = 0\%$	7.27	0.00	7.27	118.70	< .001*
CWM detailed	$FP_{comprehensible} = FP_{incomprehensible}$	8.83	14.28	5.45	0.52	= .472
CWM brief	$FP_{comprehensible} = FP_{incomprehensible}$	15.89	12.51	3.38	0.35	= .553
DQ	$FP_{comprehensible} = FP_{incomprehensible}$	1.10	7.27	6.17	5.27	= .022*
Perceived confidentiality						
Confidential	$FP_{CWM\ detailed} = FP_{CWM\ brief}$	6.36	15.97	9.61	2.03	= .154
	$FP_{CWM\ detailed} = FP_{DQ}$	6.36	1.42	4.94	0.99	= .319
	$FP_{CWM\ brief} = FP_{DQ}$	15.97	1.42	14.55	12.55	< .001*
	$FP_{CWM\ detailed} = 0\%$	6.36	0.00	6.36	1.78	= .182
	$FP_{CWM\ brief} = 0\%$	15.97	0.00	15.97	17.92	< .001*
	$FP_{CWM\ brief} = 0\%$	1.42	0.00	1.42	52.69	< .001*
Not confidential	$FP_{CWM\ detailed} = FP_{CWM\ brief}$	16.52	12.91	3.61	0.42	= .515
	$FP_{CWM\ detailed} = FP_{DQ}$	16.52	4.17	12.35	7.20	= .007*
	$FP_{CWM\ brief} = FP_{DQ}$	12.91	4.17	8.74	3.96	= .046*
	$FP_{CWM\ detailed} = 0\%$	16.52	0.00	16.52	21.56	< .001*
	$FP_{CWM\ brief} = 0\%$	12.91	0.00	12.91	14.07	< .001*

	$FP_{CWM\ brief} = 0\%$	4.17	0.00	4.17	114.11	< .001*
CWM detailed	$FP_{confidential} = FP_{not\ confidential}$	6.36	16.52	10.16	2.35	= .125
CWM brief	$FP_{confidential} = FP_{not\ confidential}$	15.97	12.91	3.06	0.29	= .592
DQ	$FP_{confidential} = FP_{not\ confidential}$	1.42	4.17	2.75	1.71	= .191
Perceived clarity						
Clear	$FP_{CWM\ detailed} = FP_{CWM\ brief}$	7.40	14.35	6.95	1.26	= .261
	$FP_{CWM\ detailed} = FP_{DQ}$	7.40	1.14	6.26	1.67	= .196
	$FP_{CWM\ brief} = FP_{DQ}$	14.35	1.14	13.21	16.26	< .001*
	$FP_{CWM\ detailed} = 0\%$	7.40	0.00	7.40	2.48	= .115
	$FP_{CWM\ brief} = 0\%$	14.35	0.00	14.35	23.16	< .001*
	$FP_{CWM\ brief} = 0\%$	1.14	0.00	1.14	51.82	< .001*
Unclear	$FP_{CWM\ detailed} = FP_{CWM\ brief}$	16.15	14.25	1.90	0.08	= .780
	$FP_{CWM\ detailed} = FP_{DQ}$	16.15	6.45	9.70	3.21	= .073
	$FP_{CWM\ brief} = FP_{DQ}$	14.25	6.45	7.80	1.53	= .215
	$FP_{CWM\ detailed} = 0\%$	16.15	0.00	16.15	20.28	< .001*
	$FP_{CWM\ brief} = 0\%$	14.25	0.00	14.25	8.54	= .003*
	$FP_{CWM\ brief} = 0\%$	6.45	0.00	6.45	117.70	< .001
CWM detailed	$FP_{clear} = FP_{unclear}$	7.40	16.15	8.75	1.77	= .184
CWM brief	$FP_{clear} = FP_{unclear}$	14.35	14.25	0.10	0.00	= .988
DQ	$FP_{clear} = FP_{unclear}$	1.14	6.45	5.31	4.44	= .035*

Note. FP = False positives.
* $p < .05$
