# Supplementary material for: Can detailed instructions and comprehension checks increase the validity of crosswise model estimates?
Source: PLoS One. 2020 Jun 30;15(6):e0235403. doi: 10.1371/journal.pone.0235403 (PMC7326177; doi:10.1371/journal.pone.0235403)
Supplement: S2 Appendix — (PDF) [file pone.0235403.s002.pdf]

Supporting Information File: Appendix B

Parameter comparisons of false negatives for the total sample and split by randomness of responses, perceived comprehensibility, perceived confidentiality and perceived clarity of the questioning technique

Parameter Comparisons: False Negatives						
					Model fit	
		Parameter 1 (in %)	Parameter 2 (in %)	|*Difference*| (in %)	ΔG^2 (df = 1)	p
Total Sample						
	$FN_{CWM\ detailed} = FN_{CWM\ brief}$	65.99	56.65	9.34	5.15	= .023*
	$FN_{CWM\ detailed} = FN_{DQ}$	65.99	81.77	15.78	18.47	< .001*
	$FN_{CWM\ brief} = FN_{DQ}$	56.65	81.77	25.12	47.71	< .001*
	$FN_{CWM\ detailed} = 0\%$	65.99	0.00	65.99	601.97	< .001*
	$FN_{CWM\ brief} = 0\%$	56.65	0.00	56.65	522.21	< .001*
	$FN_{CWM\ brief} = 0\%$	81.77	0.00	81.77	9918.94	< .001*
Randomness of responses						
Non-random	$FN_{CWM\ detailed} = FN_{CWM\ brief}$	70.94	57.76	13.18	7.73	= .005*
	$FN_{CWM\ detailed} = FN_{DQ}$	70.94	84.97	14.03	11.79	< .001*
	$FN_{CWM\ brief} = FN_{DQ}$	57.76	84.97	27.21	48.32	< .001*
	$FN_{CWM\ detailed} = 0\%$	70.94	0.00	70.94	93.447	< .001*
	$FN_{CWM\ brief} = 0\%$	57.76	0.00	57.76	440.19	< .001*
	$FN_{CWM\ brief} = 0\%$	84.97	0.00	84.97	8710.31	< .001*
Random	$FN_{CWM\ detailed} = FN_{CWM\ brief}$	54.93	51.78	3.15	0.13	= .713
	$FN_{CWM\ detailed} = FN_{DQ}$	54.93	64.82	9.89	1.33	= .250
	$FN_{CWM\ brief} = FN_{DQ}$	51.78	64.82	13.04	1.94	= .163
	$FN_{CWM\ detailed} = 0\%$	54.93	0.00	54.93	133.18	< .001*
	$FN_{CWM\ brief} = 0\%$	51.78	0.00	51.78	82.69	< .001*
	$FN_{CWM\ brief} = 0\%$	64.82	0.00	64.82	1219.40	< .001*

CWM detailed	$FN_{non\text{-}random} = FN_{random}$	70.94	54.93	16.01	6.14	= .013*
CWM brief	$FN_{non\text{-}random} = FN_{random}$	57.76	51.78	5.98	0.67	= .412
DQ	$FN_{non\text{-}random} = FN_{random}$	84.97	64.82	20.15	10.78	= .001*
Perceived comprehensibility						
Comprehensible	$FN_{CWM\ detailed} = FN_{CWM\ brief}$	72.07	58.17	13.90	3.06	= .080
	$FN_{CWM\ detailed} = FN_{DQ}$	72.07	85.20	13.13	3.65	= .056
	$FN_{CWM\ brief} = FN_{DQ}$	58.17	85.20	27.03	35.44	< .001*
	$FN_{CWM\ detailed} = 0\%$	72.07	0.00	72.07	130.22	< .001*
	$FN_{CWM\ brief} = 0\%$	58.17	0.00	58.17	289.79	< .001*
	$FN_{CWM\ brief} = 0\%$	85.20	0.00	85.20	7637.60	< .001*
Incomprehensible	$FN_{CWM\ detailed} = FN_{CWM\ brief}$	64.62	54.94	9.68	3.35	= .067
	$FN_{CWM\ detailed} = FN_{DQ}$	64.62	72.22	7.60	1.67	= .197
	$FN_{CWM\ brief} = FN_{DQ}$	54.94	72.22	17.28	7.12	= .008*
	$FN_{CWM\ detailed} = 0\%$	64.62	0.00	64.62	472.70	< .001*
	$FN_{CWM\ brief} = 0\%$	54.94	0.00	54.94	232.75	< .001*
	$FN_{CWM\ brief} = 0\%$	72.22	0.00	72.22	2288.34	< .001*
CWM detailed	$FN_{comprehensible} = FN_{incomprehensible}$	72.07	64.62	7.45	0.95	= .329
CWM brief	$FN_{comprehensible} = FN_{incomprehensible}$	58.17	54.94	3.23	0.32	= .569
DQ	$FN_{comprehensible} = FN_{incomprehensible}$	85.20	72.22	12.98	7.00	= .008*
Perceived confidentiality						
Confidential	$FN_{CWM\ detailed} = FN_{CWM\ brief}$	70.77	55.70	15.07	4.84	= .028*
	$FN_{CWM\ detailed} = FN_{DQ}$	70.77	87.03	16.26	8.14	= .004*
	$FN_{CWM\ brief} = FN_{DQ}$	55.70	87.03	31.33	38.50	< .001*
	$FN_{CWM\ detailed} = 0\%$	70.77	0.00	70.77	209.09	< .001*
	$FN_{CWM\ brief} = 0\%$	55.70	0.00	55.70	226.38	< .001*
	$FN_{CWM\ brief} = 0\%$	87.03	0.00	87.03	5788.69	< .001*
Not confidential	$FN_{CWM\ detailed} = FN_{CWM\ brief}$	63.89	57.41	6.48	1.53	= .216
	$FN_{CWM\ detailed} = FN_{DQ}$	63.89	75.48	11.59	5.23	= .022*
	$FN_{CWM\ brief} = FN_{DQ}$	57.41	75.48	18.07	11.77	< .001*
	$FN_{CWM\ detailed} = 0\%$	63.89	0.00	63.89	394.03	< .001*
	$FN_{CWM\ brief} = 0\%$	57.41	0.00	57.41	295.92	< .001*
	$FN_{CWM\ brief} = 0\%$	75.48	0.00	75.48	4137.78	< .001*

CWM detailed	$FN_{confidential} = FN_{not\ confidential}$	70.77	63.89	6.88	1.14	= .285
CWM brief	$FN_{confidential} = FN_{not\ confidential}$	55.70	57.41	1.71	0.09	= .764
DQ	$FN_{confidential} = FN_{not\ confidential}$	87.03	75.48	11.55	7.53	= .006*
Perceived clarity						
Clear	$FN_{CWM\ detailed} = FN_{CWM\ brief}$	67.48	53.96	13.52	4.48	= .034*
	$FN_{CWM\ detailed} = FN_{DQ}$	67.48	85.06	17.58	10.16	= .001*
	$FN_{CWM\ brief} = FN_{DQ}$	53.96	85.06	31.10	52.26	< .001*
	$FN_{CWM\ detailed} = 0\%$	67.48	0.00	67.48	200.32	< .001*
	$FN_{CWM\ brief} = 0\%$	53.96	0.00	53.96	307.84	< .001*
	$FN_{CWM\ brief} = 0\%$	85.06	0.00	85.06	7958.65	< .001*
Unclear	$FN_{CWM\ detailed} = FN_{CWM\ brief}$	65.29	61.51	3.78	0.41	= .523
	$FN_{CWM\ detailed} = FN_{DQ}$	65.29	70.89	5.60	0.78	= .378
	$FN_{CWM\ brief} = FN_{DQ}$	61.51	70.89	9.38	1.77	= .183
	$FN_{CWM\ detailed} = 0\%$	65.29	0.00	65.29	401.77	< .001*
	$FN_{CWM\ brief} = 0\%$	61.51	0.00	61.51	216.01	< .001*
	$FN_{CWM\ brief} = 0\%$	70.89	0.00	70.89	1967.82	< .001*
CWM detailed	$FN_{clear} = FN_{unclear}$	67.48	65.29	2.19	0.12	= .731
CWM brief	$FN_{clear} = FN_{unclear}$	53.96	61.51	7.55	1.63	= .201
DQ	$FN_{clear} = FN_{unclear}$	85.06	70.89	14.17	7.53	= .006*

Note. * $p < .05$
