# Supplementary material for: Can detailed instructions and comprehension checks increase the validity of crosswise model estimates?
Source: PLoS One. 2020 Jun 30;15(6):e0235403. doi: 10.1371/journal.pone.0235403 (PMC7326177; doi:10.1371/journal.pone.0235403)
Supplement: S1 File — MultiTree equations for the estimation of π, false positives and false negatives in a multinomial model. (PDF) [file pone.0235403.s003.pdf]

Supporting Information File: Multitree Equations

MultiTree equations for the estimation of π (Pi), false positives (FP) and false negatives (FN) in a multinomial model. Parameter p1 denotes the known probability of being born in November or December (p1 = .158) and parameter p2 denotes the known probability of being born between January and October (p2 = .842, Pötzsch, 2012). CWM_detailed = crosswise model with detailed instructions and comprehension questions, CWM_brief = crosswise model with brief instructions, DQ = direct questioning.

Estimation of π in the total sample:

```
CWM_detailed      CWM_detailed_both_true_both_false          1_Pi_CWM_detailed*p
CWM_detailed      CWM_detailed_one_true                      1_Pi_CWM_detailed*(1-p)
CWM_detailed      CWM_detailed_one_true                      (1-1_Pi_CWM_detailed)*p
CWM_detailed      CWM_detailed_both_true_both_false          (1-1_Pi_CWM_detailed)*(1-p)
CWM_brief         CWM_brief_both_true_both_false             2_Pi_CWM_brief*p
CWM_brief         CWM_brief_one_true                         2_Pi_CWM_brief*(1-p)
CWM_brief         CWM_brief_one_true                         (1-2_Pi_CWM_brief)*p
CWM_brief         CWM_brief_both_true_both_false             (1-2_Pi_CWM_brief)*(1-p)
DQ                DQ_true                                    3_Pi_DQ
DQ                DQ_false                                   (1-3_Pi_DQ)
```

Estimation of false positives among subsample of non-carriers of the sensitive attribute:

```
CWM_detailed      CWM_detailed_both_true_both_false          1_FP_CWM_detailed*p
CWM_detailed      CWM_detailed_one_true                      1_FP_CWM_detailed*(1-p)
CWM_detailed      CWM_detailed_one_true                      (1-1_FP_CWM_detailed)*p
CWM_detailed      CWM_detailed_both_true_both_false          (1-1_FP_CWM_detailed)*(1-p)
CWM_brief         CWM_brief_both_true_both_false             2_FP_CWM_brief*p
CWM_brief         CWM_brief_one_true                         2_FP_CWM_brief*(1-p)
CWM_brief         CWM_brief_one_true                         (1-2_FP_CWM_brief)*p
CWM_brief         CWM_brief_both_true_both_false             (1-2_FP_CWM_brief)*(1-p)
DQ                DQ_true                                    3_FP_DQ
DQ                DQ_false                                   (1-3_FP_DQ)
```

Estimation of false negatives among subsample of carriers of the sensitive attribute:

CWM_detailed	CWM_detailed_both_true_both_false	(1-1_FN_CWM_detailed)*p
CWM_detailed	CWM_detailed_one_true	(1-1_FN_CWM_detailed)*(1-p)
CWM_detailed	CWM_detailed_one_true	1_FN_CWM_detailed*p
CWM_detailed	CWM_detailed_both_true_both_false	1_FN_CWM_detailed*(1-p)
CWM_brief	CWM_brief_both_true_both_false	(1-2_FN_CWM_brief)*p
CWM_brief	CWM_brief_one_true	(1-2_FN_CWM_brief)*(1-p)
CWM_brief	CWM_brief_one_true	2_FN_CWM_brief*p
CWM_brief	CWM_brief_both_true_both_false	2_FN_CWM_brief*(1-p)
DQ	DQ_true	(1-3_FN_DQ)
DQ	DQ_false	3_FN_DQ
