# Supplementary material for: Can detailed instructions and comprehension checks increase the validity of crosswise model estimates?
Source: PLoS One. 2020 Jun 30;15(6):e0235403. doi: 10.1371/journal.pone.0235403 (PMC7326177; doi:10.1371/journal.pone.0235403)
Supplement: S2 File — Original instructions for the sensitive question by experimental condition. (PDF) [file pone.0235403.s004.pdf]

Original instructions used in the study:

Can detailed instructions and comprehension checks increase the validity of crosswise model estimates?

(Meisters, Hoffmann, & Musch, 2020)

CWM detailed:

	**German original**	**English translation**
Instructions	Anleitung<br>Bitte lesen Sie die folgende Erläuterung sorgfältig:<br>Wir werden Ihnen gleich zwei Aussagen (A und B) präsentieren, von denen sich eine auf die uns interessierende Fragestellung (unehrliche Antworten) bezieht. Die andere Aussage wird sich auf Ihren Geburtsmonat beziehen, welchen wir nicht kennen und auch nicht erfassen.<br>Um die Vertraulichkeit Ihrer Antwort zu schützen, sollen Sie diese Aussagen nicht einzeln beantworten, sondern lediglich angeben, ob Sie<br>- *beiden* Aussagen oder *keiner* Aussage zustimmen<br>**oder**<br>- *genau einer* Aussage (egal welcher) zustimmen.<br>Die beiden Aussagen lauten:	Instructions<br>Please read the following explanation carefully:<br>We will now present you with two statements (A and B), one of which will refer to the question of interest to us (dishonest answering). The other statement will refer to your month of birth, which we do not know and will not record.<br>To protect the confidentiality of your answer, you will not have to answer these statements individually, but you will only have to indicate whether<br>- *both* statements are *true* or *both* statements are *false*<br>**or whether**<br>- *exactly one* statement is true (irrespective of which one).<br>The two statements are:

	**Aussage A:** „Ich habe angegeben, mehr Anagramme gelöst zu haben, als ich tatsächlich gelöst habe.“<br>**Aussage B:** „Ich bin im November oder Dezember geboren.“<br><br>Die Antwortoptionen lauten:<br><br>[ ] Ich stimme *beiden* Aussagen oder *keiner* Aussage zu.<br>[ ] Ich stimme *genau einer* Aussage (egal welcher) zu.<br><br>Da wir nicht wissen können, welche der Aussagen Sie bejaht oder verneint haben, bleibt Ihre Antwort vertraulich. Aus Zahlen des Statistischen Bundesamtes kennen wir jedoch die relative Geburtenhäufigkeit pro Monat. Damit können wir ausrechnen, wie viele unehrliche Antworten insgesamt gegeben wurden, obwohl dies nicht für einzelne Teilnehmer möglich ist.<br><br>Um sicherzustellen, dass Sie diese Anleitung gelesen und verstanden haben, möchten wir Ihnen zunächst einige Verständnisfragen stellen:	**Statement A:** “On the anagram task, I claimed that I had solved more anagrams than I had actually solved”.<br>**Statement B:** “I was born in November or December”<br><br>The answer options are:<br><br>[ ] *Both* statements are *true* or *both* statements are *false*.<br>[ ] *Exactly one* statement is true (irrespective of which one).<br><br>Since we cannot know which of the statements you have affirmed or denied, your answer remains confidential. However, we know the relative birth rate per month from figures from the Federal Statistical Office. This enables us to calculate the overall rate of dishonest answers, although the honesty of responses cannot be determined for individual participants.<br><br>To ensure that you have read and understood these instructions, we would first like to ask several comprehension questions:
Comprehension questions 1-4	Verständnisfrage 1: Wir nehmen einmal an, Sie seien *im Februar* geboren. Wir nehmen außerdem an, dass Sie vorhin bei der Angabe der Anzahl gelöster Anagramme *nicht* übertrieben haben. Welche Antwort müssten Sie wählen?<br><br>[Antwortoptionen in zufälliger Reihenfolge präsentiert:]<br><br>Ich müsste antworten: „Ich stimme *beiden* Aussagen oder *keiner* Aussage zu.“	Comprehension question 1: Assuming you were born in *February*, and assuming you had *not* exaggerated on your report of the number of solved anagrams. Which answer would you have to give?<br><br>[answer options presented in random order:]<br><br>I would have to answer ‘*both* statements are *true* or *both* statements are *false*’

	Ich müsste antworten: „Ich stimme *genau einer* Aussage (egal welcher) zu.“<br><br>Zur Erinnerung - die beiden Aussagen lauten:<br>**Aussage A:** „Ich habe angegeben, mehr Anagramme gelöst zu haben, als ich tatsächlich gelöst habe.“<br>**Aussage B:** „Ich bin im November oder Dezember geboren.“<br><br>Verständnisfrage 2: Wir nehmen einmal an, Sie seien *im Dezember* geboren. Wir nehmen außerdem an, dass Sie vorhin bei der Angabe der Anzahl gelöster Anagramme übertrieben haben. Welche Antwort müssten Sie wählen?<br>[Antwortoptionen in zufälliger Reihenfolge präsentiert:]<br>Ich müsste antworten: „Ich stimme *genau einer* Aussage (egal welcher) zu.“<br>Ich müsste antworten: „Ich stimme *beiden* Aussagen oder *keiner* Aussage zu.“<br><br>Zur Erinnerung - die beiden Aussagen lauten:<br>**Aussage A:** „Ich habe angegeben, mehr Anagramme gelöst zu haben, als ich tatsächlich gelöst habe.“<br>**Aussage B:** „Ich bin im November oder Dezember geboren.“<br><br>Verständnisfrage 3: Wir nehmen einmal an, Sie seien *im November* geboren. Wir nehmen außerdem an, dass Sie vorhin bei der Angabe der Anzahl gelöster	I would have to answer ‘*exactly one* statement is true (irrespective of which one)’<br><br>Reminder - the two statements are:<br>**Statement A:** “On the anagram task, I claimed that I had solved more anagrams than I had actually solved”.<br>**Statement B:** “I was born in November or December”<br><br>Comprehension question 2: Assuming you were born in *December*, and assuming you had exaggerated on your report of the number of solved anagrams. Which answer would you have to give?<br>[answer options presented in random order:]<br>I would have to answer ‘*exactly one* statement is true (irrespective of which one)’<br>I would have to answer ‘*both* statements are *true* or *both* statements are *false*’<br><br>Reminder - the two statements are:<br>**Statement A:** “On the anagram task, I claimed that I had solved more anagrams than I had actually solved”.<br>**Statement B:** “I was born in November or December”<br><br>Comprehension question 3: “Assuming you were born in *November*, and assuming you had *not* exaggerated on your

	Anagramme *nicht* übertrieben haben. Welche Antwort müssten Sie wählen?<br><br>[Antwortoptionen in zufälliger Reihenfolge präsentiert:]<br><br>Ich müsste antworten: „Ich stimme *beiden* Aussagen oder *keiner* Aussage zu.“<br><br>Ich müsste antworten: „Ich stimme *genau einer* Aussage (egal welcher) zu.“<br><br>Zur Erinnerung - die beiden Aussagen lauten:<br><br>**Aussage A:** „Ich habe angegeben, mehr Anagramme gelöst zu haben, als ich tatsächlich gelöst habe.“<br>**Aussage B:** „Ich bin im November oder Dezember geboren.“<br><br>Verständnisfrage 4: Wir nehmen einmal an, Sie seien *im Juli* geboren. Wir nehmen außerdem an, dass Sie vorhin bei der Angabe der Anzahl gelöster Anagramme übertrieben haben. Welche Antwort müssten Sie wählen?<br><br>[Antwortoptionen in zufälliger Reihenfolge präsentiert:]<br><br>Ich müsste antworten: „Ich stimme *beiden* Aussagen oder *keiner* Aussage zu.“<br><br>Ich müsste antworten: „Ich stimme *genau einer* Aussage (egal welcher) zu.“	report of the number of solved anagrams. Which answer would you have to give?”<br><br>[answer options presented in random order:]<br><br>I would have to answer ‘*both* statements are *true* or *both* statements are *false*’<br><br>I would have to answer ‘*exactly one* statement is true (irrespective of which one)’<br><br>Reminder - the two statements are:<br><br>**Statement A:** “On the anagram task, I claimed that I had solved more anagrams than I had actually solved”.<br>**Statement B:** “I was born in November or December”<br><br>Comprehension question 4: “Assuming you were born in *July*, and assuming you had exaggerated on your report of the number of solved anagrams. Which answer would you have to give?”<br><br>[answer options presented in random order:]<br><br>I would have to answer ‘*both* statements are *true* or *both* statements are *false*’<br><br>I would have to answer ‘*exactly one* statement is true (irrespective of which one)’

	Zur Erinnerung - die beiden Aussagen lauten:<br>**Aussage A:** „Ich habe angegeben, mehr Anagramme gelöst zu haben, als ich tatsächlich gelöst habe.“<br>**Aussage B:** „Ich bin im November oder Dezember geboren.“	Reminder - the two statements are:<br>**Statement A:** “On the anagram task, I claimed that I had solved more anagrams than I had actually solved”.<br>**Statement B:** “I was born in November or December”
Feedback on the comprehension questions 1-4	Rückmeldung zu den Verständnisfragen<br>[Beispiel-Feedback für einen Teilnehmer, der die erste und dritte Verständnisfrage richtig, die zweite und vierte Verständnisfrage falsch beantwortet hat:]<br><br>Zur Erinnerung - die beiden Aussagen lauteten:<br>**Aussage A:** „Ich habe angegeben, mehr Anagramme gelöst zu haben, als ich tatsächlich gelöst habe.“<br>**Aussage B:** „Ich bin im November oder Dezember geboren.“<br><br>Die Antwortoptionen lauteten:<br>[ ] Ich stimme *beiden* Aussagen oder *keiner* Aussage zu.<br>[ ] Ich stimme *genau einer* Aussage (egal welcher) zu.<br><br>Verständnisfrage 1 lautete:<br>Wir nehmen einmal an, Sie seien *im Februar* geboren. Wir nehmen außerdem an, dass Sie vorhin bei der Angabe der Anzahl gelöster Anagramme *nicht* übertrieben haben. Welche Antwort müssten Sie wählen?	Feedback on the comprehension questions<br>[Example feedback for a participant who answered the first and third comprehension questions correctly and the second and fourth comprehension questions incorrectly:]<br><br>Reminder - the two statements were:<br>**Statement A:** “On the anagram task, I claimed that I had solved more anagrams than I had actually solved”.<br>**Statement B:** “I was born in November or December”<br><br>The answer options were:<br>[ ] *Both* statements are *true* or *both* statements are *false*.<br>[ ] *Exactly one* statement is true (irrespective of which one).<br><br>Comprehension question 1 was:<br>Assuming you were born in *February*, and assuming you had *not* exaggerated on your report of the number of solved anagrams. Which answer would you have to give?

	Sie haben geantwortet: „Ich stimme *beiden* Aussagen oder *keiner* Aussage zu.“<br><br>**Ihre Antwort ist richtig, da Sie weder Aussage A noch Aussage B zustimmen würden.**<br><br><br>Verständnisfrage 2 lautete:<br><br>Wir nehmen einmal an, Sie seien *im Dezember* geboren. Wir nehmen außerdem an, dass Sie vorhin bei der Angabe der Anzahl gelöster Anagramme übertrieben haben. Welche Antwort müssten Sie wählen?<br><br>Sie haben geantwortet: „Ich stimme *genau einer* Aussage (egal welcher) zu.“<br><br>**Ihre Antwort ist falsch. Sie würden sowohl Aussage A als auch Aussage B zustimmen, also wäre die richtige Antwort: „Ich stimme *beiden* Aussagen oder *keiner* Aussage zu.“**<br><br><br>Verständnisfrage 3 lautete:<br><br>Wir nehmen einmal an, Sie seien *im November* geboren. Wir nehmen außerdem an, dass Sie vorhin bei der Angabe der Anzahl gelöster Anagramme *nicht* übertrieben haben. Welche Antwort müssten Sie wählen?<br><br>Sie haben geantwortet: „Ich stimme *genau einer* Aussage (egal welcher) zu.“	Your answer was: “*Both* statements are *true* or *both* statements are *false*.”<br><br>**Your answer is correct because you would not agree to either statement A or statement B.**<br><br><br>Comprehension question 2 was:<br><br>Assuming you were born in *December*, and assuming you had exaggerated on your report of the number of solved anagrams. Which answer would you have to give?<br><br>Your answer was: “*Exactly one* of the statements is true (irrespective of which one).”<br><br>**Your answer is incorrect. You would agree with both statement A and statement B, so the correct answer would be: “*Both* statements are *true* or *both* statements are *false*”**<br><br><br>Comprehension question 3 was:<br><br>Assuming you were born in *November*, and assuming you had *not* exaggerated on your report of the number of solved anagrams. Which answer would you have to give?<br><br>Your answer was: “*Exactly one* of the statements is true (irrespective of which one).”

	**Ihre Antwort ist richtig, da Sie Aussage A ablehnen und Aussage B zustimmen würden.**<br><br>Verständnisfrage 4 lautete:<br><br>Wir nehmen einmal an, Sie seien *im Juli* geboren. Wir nehmen außerdem an, dass Sie vorhin bei der Angabe der Anzahl gelöster Anagramme übertrieben haben. Welche Antwort müssten Sie wählen?<br><br>Sie haben geantwortet: „Ich stimme *beiden* Aussagen oder *keiner* Aussage zu."<br><br>**Ihre Antwort ist falsch. Sie würden Aussage A zustimmen und Aussage B ablehnen, also wäre die richtige Antwort: „Ich stimme *genau einer* Aussage (egal welcher) zu."**<br><br>[Falls mindestens eine Verständnisfrage falsch beantwortet wurde:]<br><br>**Sie haben bei der Bearbeitung der Verständnisfragen mindestens einen Fehler gemacht. Auf der nächsten Seite werden wir Ihnen noch einmal die Verständnisfrage(n) präsentieren, die Sie bisher nicht richtig beantwortet haben. Bitte geben Sie sich Mühe, die richtige Antwort zu finden!**	**Your answer is correct because you would reject statement A and agree with statement B.**<br><br>Comprehension question 4 was:<br><br>Assuming you were born in *July*, and assuming you had exaggerated on your report of the number of solved anagrams. Which answer would you have to give?<br><br>Your answer was: "*Both* statements are *true* or *both* statements are *false*."<br><br>**Your answer is incorrect. You would agree with statement A and disagree with statement B, so the correct answer would be: "*Exactly one* of the statements is true (irrespective of which one)".**<br><br>[If at least one comprehension question was answered incorrectly:]<br><br>**You have made at least one mistake in answering the comprehension questions. On the next page, we will again present the comprehension question(s) you did not answer correctly so far. Please do your best to find the correct answer!**

	[Falls alle Verständnisfragen korrekt beantwortet wurden:]<br><br>**Prima, Sie haben alle Verständnisfragen richtig beantwortet!**	[If all comprehension questions were answered correctly:]<br><br>**Great, you have answered all comprehension questions correctly!**
Repeated presentation of instructions	Anleitung<br><br>Bitte lesen Sie sich noch einmal die folgende Erläuterung durch:<br><br>Wir werden Ihnen gleich zwei Aussagen (A und B) präsentieren, von denen sich eine auf die uns interessierende Fragestellung (unehrliche Antworten) bezieht. Die andere Aussage wird sich auf Ihren Geburtsmonat beziehen, welchen wir nicht kennen und auch nicht erfassen.<br><br>Um die Vertraulichkeit Ihrer Antwort zu schützen, sollen Sie diese Aussagen nicht einzeln beantworten, sondern lediglich angeben, ob Sie<br><br>- *beiden* Aussagen oder *keiner* Aussage zustimmen<br>**oder**<br>- *genau einer* Aussage (egal welcher) zustimmen.<br><br>Die beiden Aussagen lauten:<br><br>**Aussage A:** „Ich habe angegeben, mehr Anagramme gelöst zu haben, als ich tatsächlich gelöst habe.“<br>**Aussage B:** „Ich bin im November oder Dezember geboren.“<br><br>Die Antwortoptionen lauten:	Instructions<br><br>Please read the following explanation carefully again:<br><br>We will now present you with two statements (A and B), one of which will refer to the question of interest to us (dishonest answering). The other statement will refer to your month of birth, which we do not know and will not record.<br><br>To protect the confidentiality of your answer, you will not have to answer these statements individually, but you will only have to indicate whether<br><br>- *both* statements are *true* or *both* statements are *false*<br>**or whether**<br>- *exactly one* statement is true (irrespective of which one).<br><br>The two statements are:<br><br>**Statement A:** “On the anagram task, I claimed that I had solved more anagrams than I had actually solved”.<br>**Statement B:** “I was born in November or December”<br><br>The answer options are:

	[ ] Ich stimme beiden Aussagen oder keiner Aussage zu.<br>[ ] Ich stimme genau einer Aussage (egal welcher) zu.<br><br>Da wir nicht wissen können, welche der Aussagen Sie bejaht oder verneint haben, bleibt Ihre Antwort vertraulich. Aus Zahlen des Statistischen Bundesamtes kennen wir jedoch die relative Geburtenhäufigkeit pro Monat. Damit können wir ausrechnen, wie viele unehrliche Antworten insgesamt gegeben wurden, obwohl dies nicht für einzelne Teilnehmer möglich ist.<br><br>Um sicherzustellen, dass Sie diese Anleitung gelesen und verstanden haben, beantworten Sie bitte noch diese beiden Verständnisfragen:	[ ] *Both* statements are *true* or *both* statements are *false*.<br>[ ] *Exactly one* statement is true (irrespective of which one).<br><br>Since we cannot know which of the statements you have affirmed or denied, your answer remains confidential. However, we know the relative birth rate per month from figures from the Federal Statistical Office. This enables us to calculate the overall rate of dishonest answers, although the honesty of responses cannot be determined for individual participants.<br><br>To ensure that you have read and understood these instructions, please also answer these two comprehension questions:
Comprehension questions 5-6	Verständnisfrage 5: Stellen Sie sich vor, Sie hätten die Option gewählt 'Ich stimme *beiden* Aussagen oder *keiner* Aussage zu.' Was könnte jemand, der Ihren Geburtsmonat nicht kennt, aus dieser Wahl folgern?<br><br>- Er könnte folgern, dass Sie bei der Angabe der Anzahl gelöster Anagramme übertrieben haben.<br><br>- Er könnte folgern, dass Sie die Zahl gelöster Anagramme korrekt angegeben haben.<br><br>- Er könnte daraus weder das eine noch das andere folgern.<br><br>Zur Erinnerung - die beiden Aussagen lauten:<br><br>**Aussage A:** „Ich habe angegeben, mehr Anagramme gelöst zu haben, als ich tatsächlich gelöst habe.“	Comprehension question 5: Imagine you had chosen the option ‘*Both* statements are *true* or *both* statements are *false*’. What could someone who does not know your birth month infer from your choice?<br><br>- He could infer that you had exaggerated your report of the number of solved anagrams.<br><br>- He could infer that you reported the number of solved anagrams truthfully.<br><br>- He could infer none of the above.<br><br>Reminder - the two statements are:<br><br>**Statement A:** “On the anagram task, I claimed that I had solved more anagrams than I had actually solved”.

	**Aussage B:** „Ich bin im November oder Dezember geboren.“<br><br>Verständnisfrage 6: Stellen Sie sich vor, Sie hätten die Option gewählt 'Ich stimme *genau einer* Aussage (egal welcher) zu.' Was könnte jemand, der Ihren Geburtsmonat nicht kennt, aus dieser Wahl folgern?<br>- Er könnte folgern, dass Sie bei der Angabe der Anzahl gelöster Anagramme übertrieben haben.<br>- Er könnte folgern, dass Sie die Zahl gelöster Anagramme korrekt angegeben haben.<br>- Er könnte daraus weder das eine noch das andere folgern.<br><br>Zur Erinnerung - die beiden Aussagen lauten:<br>**Aussage A:** „Ich habe angegeben, mehr Anagramme gelöst zu haben, als ich tatsächlich gelöst habe.“<br>**Aussage B:** „Ich bin im November oder Dezember geboren.“	**Statement B:** “I was born in November or December”<br><br>Comprehension question 6: Imagine you had chosen the option ‘*Exactly one* statement is true (irrespective of which one)’. What could someone who does not know your birth month infer from your choice?<br>- He could infer that you had exaggerated your report of the number of solved anagrams.<br>- He could infer that you reported the number of solved anagrams truthfully.<br>- He could infer none of the above.<br><br>Reminder - the two statements are:<br>**Statement A:** “On the anagram task, I claimed that I had solved more anagrams than I had actually solved”.<br>**Statement B:** “I was born in November or December”
Feedback on comprehension questions 5-6	Rückmeldung zu den Verständnisfragen<br>[Beispiel-Feedback für einen Teilnehmer, der die fünfte Verständnisfrage falsch und die sechste Verständnisfrage richtig beantwortet hat:]<br><br>Zur Erinnerung - die beiden Aussagen lauteten:	Feedback on the comprehension questions<br>[Example feedback for a participant who answered the fifth comprehension question incorrectly and the sixth comprehension questions correctly:]<br><br>Reminder - the two statements were:

	**Aussage A:** „Ich habe angegeben, mehr Anagramme gelöst zu haben, als ich tatsächlich gelöst habe.“<br>**Aussage B:** „Ich bin im November oder Dezember geboren.“<br><br>Die Antwortoptionen lauteten:<br>[ ] Ich stimme *beiden* Aussagen oder *keiner* Aussage zu.<br>[ ] Ich stimme *genau einer* Aussage (egal welcher) zu.<br><br>Verständnisfrage 5 lautete:<br>Stellen Sie sich vor, Sie hätten die Option gewählt 'Ich stimme *beiden* Aussagen oder *keiner* Aussage zu.' Was könnte jemand, der Ihren Geburtsmonat nicht kennt, aus dieser Wahl folgern?<br>Sie haben geantwortet: "Er könnte folgern, dass Sie die Zahl gelöster Anagramme korrekt angegeben haben."<br>**Ihre Antwort ist falsch. Sie könnten entweder angegeben haben, bei der Angabe der Anzahl gelöster Anagramme übertrieben zu haben, und im November oder Dezember geboren zu sein, oder die Zahl gelöster Anagramme korrekt angegeben zu haben, und zwischen Januar und Oktober geboren zu sein. Also wäre die richtige Antwort: „Er könnte daraus weder das eine noch das andere folgern.“**	**Statement A:** “On the anagram task, I claimed that I had solved more anagrams than I had actually solved”.<br>**Statement B:** “I was born in November or December”<br><br>The answer options were:<br>[ ] *Both* statements are *true* or *both* statements are *false*.<br>[ ] *Exactly one* statement is true (irrespective of which one).<br><br>Comprehension question 5 was:<br>Imagine you had chosen the option ‘*Both* statements are *true* or *both* statements are *false*’. What could someone who does not know your birth month infer from your choice?<br>Your answer was: ‘He could infer that you reported the number of solved anagrams truthfully.’<br>**Your answer is incorrect. You may have either stated that you exaggerated on the number of anagrams solved and that you were born in November or December, or that you correctly stated the number of anagrams solved and that you were born between January and October. So the correct answer would be: He could infer none of the above.**

	Verständnisfrage 6 lautete:<br><br>Stellen Sie sich vor, Sie hätten die Option gewählt 'Ich stimme *genau einer* Aussage (egal welcher) zu.' Was könnte jemand, der Ihren Geburtsmonat nicht kennt, aus dieser Wahl folgern?<br><br>Sie haben geantwortet: "Er könnte daraus weder das eine noch das andere folgern."<br><br>**Ihre Antwort ist richtig. Sie könnten entweder angegeben haben, bei der Angabe der Anzahl gelöster Anagramme übertrieben zu haben, und zwischen Januar und Oktober geboren zu sein, oder die Zahl gelöster Anagramme korrekt angegeben zu haben, und im November oder Dezember geboren zu sein.**<br><br>[Falls mindestens eine Verständnisfrage falsch beantwortet wurde:]<br><br>**Sie haben bei der Bearbeitung der Verständnisfragen mindestens einen Fehler gemacht. Auf der nächsten Seite werden wir Ihnen noch einmal die Verständnisfrage(n) präsentieren, die Sie bisher nicht richtig beantwortet haben. Bitte geben Sie sich Mühe, die richtige Antwort zu finden!**<br><br>[Falls alle Verständnisfragen korrekt beantwortet wurden:]<br><br>**Prima, Sie haben alle Verständnisfragen richtig beantwortet!**	Comprehension question 6 was:<br><br>Imagine you had chosen the option '*Exactly one* statement is true (irrespective of which one)'. What could someone who does not know your birth month infer from your choice?<br><br>Your answer was: 'He could infer none of the above.'<br><br>**Your answer is correct. You may have either stated that you exaggerated on the number of anagrams solved and that you were born between January and October, or that you correctly stated the number of anagrams solved and that you were born in November or December.**<br><br>[If at least one comprehension question was answered incorrectly:]<br><br>**You have made at least one mistake in answering the comprehension questions. On the next page, we will again present the comprehension question(s) you did not answer correctly so far. Please do your best to find the correct answer!**<br><br>[If all comprehension questions were answered correctly:]<br><br>**Great, you have answered all comprehension questions correctly!**

Sensitive question	Nun sind wir an Ihrer wirklichen, auf Sie persönlich zutreffenden Antwort interessiert. Denken Sie daran:<br>**Wir kennen Ihren Geburtsmonat nicht und werden diesen auch nicht erfassen!**<br>Die Vertraulichkeit Ihrer Antwort ist somit gewährleistet. Im Folgenden finden Sie noch einmal die Anleitung zur Beantwortung der Frage:<br><br>Anleitung<br>Wir werden Ihnen gleich zwei Aussagen (A und B) präsentieren, von denen sich eine auf die uns interessierende Fragestellung (unehrliche Antworten) bezieht. Die andere Aussage wird sich auf Ihren Geburtsmonat beziehen, welchen wir nicht kennen und auch nicht erfassen.<br>Um die Vertraulichkeit Ihrer Antwort zu schützen, sollen Sie diese Aussagen nicht einzeln beantworten, sondern lediglich angeben, ob Sie<br>- *beiden* Aussagen oder *keiner* Aussage zustimmen<br>**oder**<br>- *genau einer* Aussage (egal welcher) zustimmen.<br>Die beiden Aussagen lauten:<br>**Aussage A:** „Ich habe angegeben, mehr Anagramme gelöst zu haben, als ich tatsächlich gelöst habe.“	Now we are interested in your real answer that is personally applicable to you. Remember:<br>**We do not know your month of birth and we will not record it!**<br>The confidentiality of your answer is therefore guaranteed. In the following you will find the instructions for answering the question once again:<br><br>Instructions<br>We will now present you with two statements (A and B), one of which will refer to the question of interest to us (dishonest answering). The other statement will refer to your month of birth, which we do not know and will not record.<br>To protect the confidentiality of your answer, you will not have to answer these statements individually, but you will only have to indicate whether<br>- *both* statements are *true* or *both* statements are *false*<br>**or whether**<br>- *exactly one* statement is true (irrespective of which one).<br>The two statements are:<br>**Statement A:** “On the anagram task, I claimed that I had solved more anagrams than I had actually solved”.

	**Aussage B:** „Ich bin im November oder Dezember geboren.“<br><br>Da wir nicht wissen können, welche der Aussagen Sie bejaht oder verneint haben, bleibt Ihre Antwort vertraulich. Aus Zahlen des Statistischen Bundesamtes kennen wir jedoch die relative Geburtenhäufigkeit pro Monat. Damit können wir ausrechnen, wie viele unehrliche Antworten insgesamt gegeben wurden, obwohl dies nicht für einzelne Teilnehmer möglich ist.<br><br>Bitte nehmen Sie nun Stellung zu den beiden Aussagen:<br><br>- Ich stimme *beiden* Aussagen oder *keiner* Aussage zu.<br><br>- Ich stimme *genau einer* Aussage (egal welcher) zu.	**Statement B:** “I was born in November or December”<br><br>Since we cannot know which of the statements you have affirmed or denied, your answer remains confidential. However, we know the relative birth rate per month from figures from the Federal Statistical Office. This enables us to calculate the overall rate of dishonest answers, although the honesty of responses cannot be determined for individual participants.<br><br>Please respond to the two statements now:<br><br>- *Both* statements are *true* or *both* statements are *false*.<br><br>- *Exactly one* statement is true (irrespective of which one).

CWM brief:

	**German original**	**English translation**
Sensitive question	Anleitung<br><br>Wir werden Ihnen gleich zwei Aussagen (A und B) präsentieren, von denen sich eine auf die uns interessierende Fragestellung (unehrliche Antworten) bezieht. Die andere Aussage wird sich auf Ihren Geburtsmonat beziehen, welchen wir nicht kennen und auch nicht erfassen.<br><br>Um die Vertraulichkeit Ihrer Antwort zu schützen, sollen Sie diese Aussagen nicht einzeln beantworten, sondern lediglich angeben, ob Sie<br><br>- *beiden* Aussagen oder *keiner* Aussage zustimmen<br>**oder**<br>- *genau einer* Aussage (egal welcher) zustimmen.<br><br>Die beiden Aussagen lauten:<br><br>**Aussage A:** „Ich habe angegeben, mehr Anagramme gelöst zu haben, als ich tatsächlich gelöst habe.“<br>**Aussage B:** „Ich bin im November oder Dezember geboren.“<br><br>Bitte nehmen Sie nun Stellung zu den beiden Aussagen:<br><br>- Ich stimme *beiden* Aussagen oder *keiner* Aussage zu.<br><br>- Ich stimme *genau einer* Aussage (egal welcher) zu.	Instructions<br><br>We will now present you with two statements (A and B), one of which will refer to the question of interest to us (dishonest answering). The other statement will refer to your month of birth, which we do not know and will not record.<br><br>To protect the confidentiality of your answer, you will not have to answer these statements individually, but you will only have to indicate whether<br><br>- *both* statements are *true* or *both* statements are *false*<br>**or whether**<br>- *exactly one* statement is true (irrespective of which one).<br><br>The two statements are:<br><br>**Statement A:** “On the anagram task, I claimed that I had solved more anagrams than I had actually solved”.<br>**Statement B:** “I was born in November or December”<br><br>Please respond to the two statements now:<br><br>- *Both* statements are *true* or *both* statements are *false.*<br><br>- *Exactly one* statement is true (irrespective of which one).

DQ:

	**German original**	**English translation**
Sensitive question	**Aussage:** „Ich habe angegeben, mehr Anagramme gelöst zu haben, als ich tatsächlich gelöst habe.“<br>- Stimmt.<br>- Stimmt nicht.	**Statement:** “On the anagram task, I claimed that I had solved more anagrams than I had actually solved”.<br>- True<br>- False
