# Supplementary material for: Can detailed instructions and comprehension checks increase the validity of crosswise model estimates?
Source: PLoS One. 2020 Jun 30;15(6):e0235403. doi: 10.1371/journal.pone.0235403 (PMC7326177; doi:10.1371/journal.pone.0235403)
Supplement: S1 Data — (PDF) [file pone.0235403.s005.pdf]

Supporting Information File: Data

Empirically observed answer frequencies for the attributes used for parameter estimation in multiTree (Moshagen, 2010) version 0.46. CWM_detailed = crosswise model with detailed instructions and comprehension questions, CWM_brief = crosswise model with brief instructions, DQ = direct questioning.

Total Sample

```
CWM_detailed_both_true_both_false       649
CWM_detailed_one_true                   323
CWM_brief_both_true_both_false          735
CWM_brief_one_true                      429
DQ_true                                 68
DQ_false                                509
```

Only non-carriers of the sensitive attribute (required for estimation of false positives)

```
CWM_detailed_both_true_both_false       298
CWM_detailed_one_true                   98
CWM_brief_both_true_both_false          375
CWM_brief_one_true                      129
DQ_true                                 6
DQ_false                                231
```

Only carriers of the sensitive attribute (required for estimation of false negatives)

```
CWM_detailed_both_true_both_false       351
```

CWM_detailed_one_true	225
CWM_brief_both_true_both_false	360
CWM_brief_one_true	300
DQ_true	62
DQ_false	278
